# Multilocus DNA barcoding – Species Identification with Multilocus Data

**DOI:** 10.1038/s41598-017-16920-2

**Published:** 2017-11-30

**Authors:** Junning Liu, Jiamei Jiang, Shuli Song, Luke Tornabene, Ryan Chabarria, Gavin J. P. Naylor, Chenhong Li

**Affiliations:** 1 Shanghai Universities Key Laboratory of Marine Animal Taxonomy and Evolution, Shanghai, 201306 China; 2 Key Laboratory of Exploration and Utilization of Aquatic Genetic Resources, Shanghai Ocean University, Ministry of Education, Shanghai, 201306 China; 3 National Demonstration Center for Experimental Fisheries Science Education (Shanghai Ocean University), Shanghai, 201306 China; 4 School of Aquatic and Fisheries Sciences, University of Washington, Seattle, WA 98195 USA; 5 College of Science & Engineering, Texas A&M University – Corpus Christi, Corpus Christi, TX 78412-5806 USA; 6 University of Florida, Gainesville, FL 3261 USA

## Abstract

Species identification using DNA sequences, known as DNA barcoding has been widely used in many applied fields. Current barcoding methods are usually based on a single mitochondrial locus, such as cytochrome c oxidase subunit I (COI). This type of barcoding method does not always work when applied to species separated by short divergence times or that contain introgressed genes from closely related species. Herein we introduce a more effective multi-locus barcoding framework that is based on gene capture and “next-generation” sequencing. We selected 500 independent nuclear markers for ray-finned fishes and designed a three-step pipeline for multilocus DNA barcoding. We applied our method on two exemplar datasets each containing a pair of sister fish species: *Siniperca chuatsi* vs. *Sini*. *kneri* and *Sicydium altum* vs. *Sicy*. *adelum*, where the COI barcoding approach failed. Both of our empirical and simulated results demonstrated that under limited gene flow and enough separation time, we could correctly identify species using multilocus barcoding method. We anticipate that, as the cost of DNA sequencing continues to fall that our multilocus barcoding approach will eclipse existing single-locus DNA barcoding methods as a means to better understand the diversity of the living world.

## Introduction

DNA barcoding has been very successfully employed in many applied fields, ranging from routine species identification^1–3^, to discovery of cryptic species^4,5^, tracking of invasive species^6–8^, conservation, and community ecology^9–12^. The mitochondrial cytochrome c oxidase subunit I gene (COI) has a good amount of variation and is easy to amplify using PCR based approaches in most animal groups^13–16^. It has become the most commonly used marker for animal DNA barcoding since it was first proposed more than a decade ago^13^. In most cases, single-locus (COI) DNA barcoding results in successful species identification. For example, a success rate close to 100% were reported for Germany herpetofauna^17^, more than 90% for Chinese rodents^18^, more than 80% for freshwater fishes of the Congo basin^19,20^, and 100% for mosquitoes^21^. However, the success rate of species identification was low for species complexes with gene flow^17^ or where species had only recently diverged^22^.

In order to use barcoding for species identification, within species variation must be less than between species variation. This generates a “break” in the distribution of distances that is referred to as the “barcoding gap”. Indeed one of the common causes of barcoding failure occurs when differences in demography eliminate the barcoding gap, because intra-specific differences are greater than inter-specific differences for the clades being compared. To an extreme, two individuals could have the same COI sequence, while being distinctly different species. Shared COI haplotypes have been reported in different species of spiders^23^, birds^24^ and fishes^25^. The single-locus barcoding is prone to misidentification when different species share haplotypes.

Although haplotypes at a single locus, such as COI can be shared between two species, it is unlikely that individuals of two species share alleles across multiple independent genes. Accordingly, multilocus data should perform better for species identification than any single locus could. Dowton *et al*.^26^ proposed “next-generation” DNA barcoding based on multilocus data in which they incorporated multispecies coalescent species delimitation. They analyzed *Sarcophaga* flesh flies with two loci, mitochondrial COI and nuclear carbomoylphosphate synthase (CAD), and found out that their coalescent-based *BEAST/BPP approach was more successful than standard barcoding method^26^. However, Collins and Cruickshank^27^ reanalyzed Dowton *et al*.’s data and showed that standard single locus (COI) barcoding method could achieve the same accuracy as the new multilocus framework did if an optimized distance threshold was applied^28–31^. The experiment of Dowton *et al*.^26^ seems unsuccessful, but the likely reason for this is that the data they used was not challenging enough for standard single-locus barcoding methods, because there was only one unidentifiable individual that was more divergent from its closest putative conspecific than the optimized threshold^27^. The other reason is that only a single nuclear gene was used in their study, thus provided little additional information^27^.

In the past it has been challenging to obtain sequences from sufficient independent nuclear loci from a broad taxonomic group to make multilocus DNA barcoding effective. Tools for finding thousands of nuclear gene markers^32–34^ and collecting their sequences through cross-species gene capture and next-generation sequencing are now available^35^, providing an opportunity to rigorously test the power of multilocus DNA barcoding. In this study, we screened for hundreds of nuclear gene markers for ray-finned fish and developed a three-step procedure for species identification. We tested our multilocus DNA barcodes in both empirical and simulated data. Our goal is to develop a multilocus barcoding approach for identifying species that are indistinguishable based on the current DNA barcoding method.

## Results

We first investigated effect of increasing number of loci on species discrimination using empirical data (between *Siniperca chuatsi* and *Sini*. *kneri* and between *Sicydium altum* and *Sicy*. *adelum*). We subsequently estimated the population parameters, gene flow and divergence time for both pairs of species. Guided by the patterns seen in the empirical data, we simulated sequences with different splitting times and migration rates, and explored the effect of divergence time and gene flow on the success rate of species identification over a broader range of the relevant parameter space. Finally, we selected 500 nuclear markers for ray-finned fishes, designed a three-step pipeline for multilocus DNA barcoding and tested the new method on species identification.

### Species discrimination using empirical data

We have developed 4,434 single-copy nucleotide loci for ray-finned fishes, and tested them in 83 species (33 families and 11 orders), covering major clades of ray-finned fishes^36^. Those markers have few missing data in the taxa tested, showing promise for their deployment in phylogenetics and population genetic analyses. We adopted those 4,434 loci as candidate barcoding markers in order to further optimize a subset of universal markers for all ray-finned fishes. We choose loci that could be readily captured and sequenced across taxa, and that were variable based on their average p-distance values among taxa.

Some of the most challenging instances for DNA barcoding occur when taxa are recently diverged or when gene flow exists between closely related species, or both. In an effort to design a rigorous barcoding scheme, we picked empirical study systems that would involve both challenges. The first involved sinipercid fishes, a family of fishes containing two genera, 9 to 12 species depending on the authority referenced^37–40^. Among them, two sister species, *Siniperca chuatsi* and *Sini*. *kneri* have distinct morphological characters, such as number of plyoriccaecum, ratio between eye length and head length^39^, but they are not distinguishable using mitochondrial control region sequences^41^. These two sister species are allopatric in most of their distribution regions^39,40^, so the reason for unsuccessful species identification in these sister species is likely due to their recency of speciation^41^. The other group of fishes that we checked is *Sicydium*. *Sicydium* is a group of diadromous gobies native to fast-flowing streams and rivers of the Americas (Central America, Mexico, Cocos Island, the Caribbean, Colombia, Ecuador and Venezuela) and Africa. There are two syntopic species, *Sicy*. *altum* and *Sicy*. *adelum* that could be separated according to distinct dental papillae and other morphological characters^42^, but they are indistinguishable using mitochondrial or nuclear genes^43^. Because these two closely related species are frequently found together^42^, it is possible that they have been subject to interspecific gene flow which would account for the high degree of genetic similarity between them. These two pairs of sister-species were used as test cases to evaluate how gene flow and shallow divergence times might affect species discrimination and identification based on multilocus barcoding.

**Figure 1:**
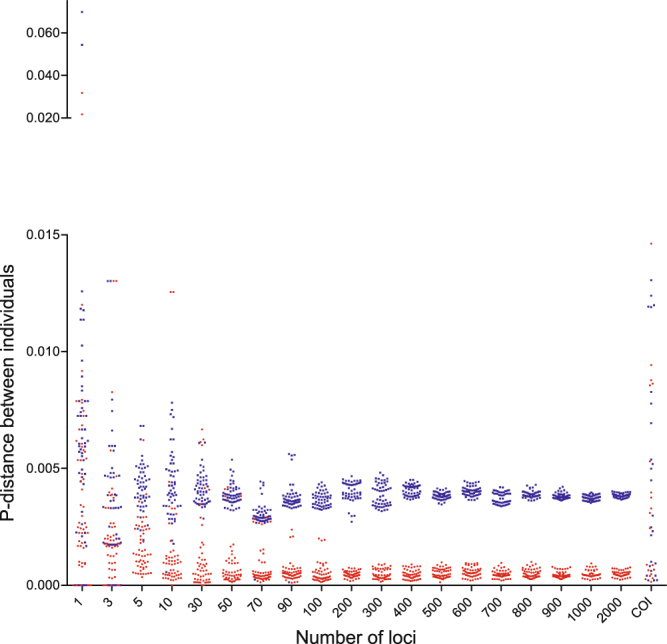
Intra- (red) and interspecific (blue) p-distance of *Siniperca chuatsi* and *Sini*. *kneri* calculated using different number of nuclear loci or COI gene. Scale of distances larger than 0.020 was reduced to fit all data points in the art board.

After all loci with missing taxa were excluded, 2,586 loci were retained for *Siniperca*. The intra- and interspecific p-distances between five individuals of *Sini*. *chuatsi* and five *Sini*. *kneri* using different numbers of nuclear loci or COI are shown in Fig. 1. The intraspecific p-distance (red) calculated using one locus or a small number of loci overlap with interspecific p-distance (blue). There is no barcoding gap separating the intra- and interspecific distances. Intraspecific distances did not become distinguishable from interspecific distances until more than 90 loci were used. The gap separating the intra- and interspecific distance increased as more loci were added, but had little effect after 400 loci were used. The variance of the intra- and interspecific p-distance decreased when more loci were included in calculating the p-distance. The p-distance calculated on COI sequences had overlapped intra- and interspecific values, so that the mean intraspecific distance was 0.0462 (0.0025 – 0.1542) and the mean interspecific distance was 0.0539 (0.0025–0.1347) (Fig. 1).

**Figure 2:**
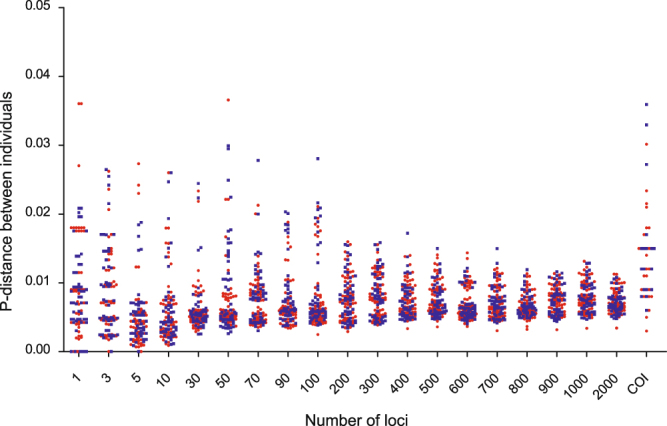
Intra- (red) and interspecific (blue) p-distance of *Sicydium altum* and *Sicy*. *adelum* calculated using different numbers of nuclear loci or COI gene.

Similar p-distance calculations on *Sicy*. *altum* and *Sicy*. *adelum* resulted in a different pattern than the one observed for *Siniperca*. The intra- (red) and interspecific (blue) p-distances in *Sicydium* were always mixed together, no matter how many loci were included in the analysis. The variance of intra- and interspecific p-distance decreased when more loci were included. The intra- and interspecific p-distances calculated using COI also were indistinguishable (Fig. 2).

**Figure 3:**
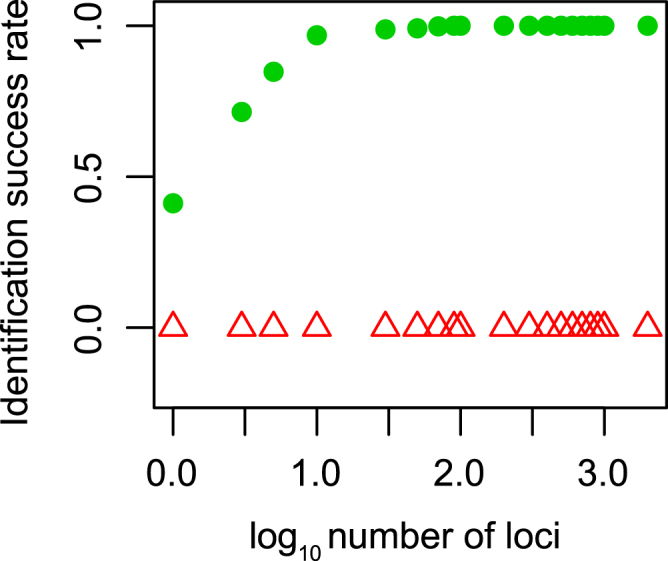
The relationship between number of loci used and success rate of identification between *Siniperca chuatsi* and *Sini*. *kneri* (green dots), and between *Sicydium altum* and *Sicy*. *adelum* (red triangles).

The success rate of identification was low (0.412) in *Siniperca* when only one locus was used based on “all species barcodes” criterion^44^ with two hundred trials, but it rose up quickly and reached 1.0 after more than 90 loci were added to the dataset (green dots, Fig. 3; Supplementary Table S1). The identification success rate was zero in *Sicydium* according to the “all species barcodes” criterion, no matter how many loci were included in the analysis (red triangles, Fig. 3). We also applied the COI barcoding approach with an optimized threshold^28^. The success rate of species identification using COI was zero in both *Siniperca* and *Sicydium*.

### Population parameters inferred for the two species pairs

To investigate the difference seen in the results of the *Siniperca* and *Sicydium* analyses, we explored some of the population attributes associated with each of these two groups. Structure analysis showed that K equaled to 2 had the highest probability when analyzing the two *Siniperca* species (Supplementary Fig. S1), but the two *Sicydium* species were indistinguishable (Supplementary Fig. S1). The divergence time between *Sini*. *chuatsi* and *Sini*. *kneri* was estimated as t_0_ = 1.754, which would be equal to ~800,000 generations if we assume an average locus size of 300 bp, a generation time of 2–3 years for *Siniperca* and a substitution rate of 2.22 × 10^−9^ per site per year^45^. Gene flow from *Sini*. *chuatsi* to *Sini*. *kneri* was 0.157 (not significant by LLRtest), but gene flow from *Sini*. *kneri* to *Sini*. *chuatsi* was highly significant, 0.640 (p < 0.001). The divergence time between *Sicy*. *altum* and *Sicy*. *adelum* was estimated as t_0_ = 0.003195, which was not significantly different from zero (HPD95_Lo_ = 0). Gene flow from *Sicy*. *altum* to *Sicy*. *adelum* was 0.494, and gene flow from *Sicy*. *adelum* to *Sicy*. *altum* was 0.502.

### Simulation results

**Figure 4:**
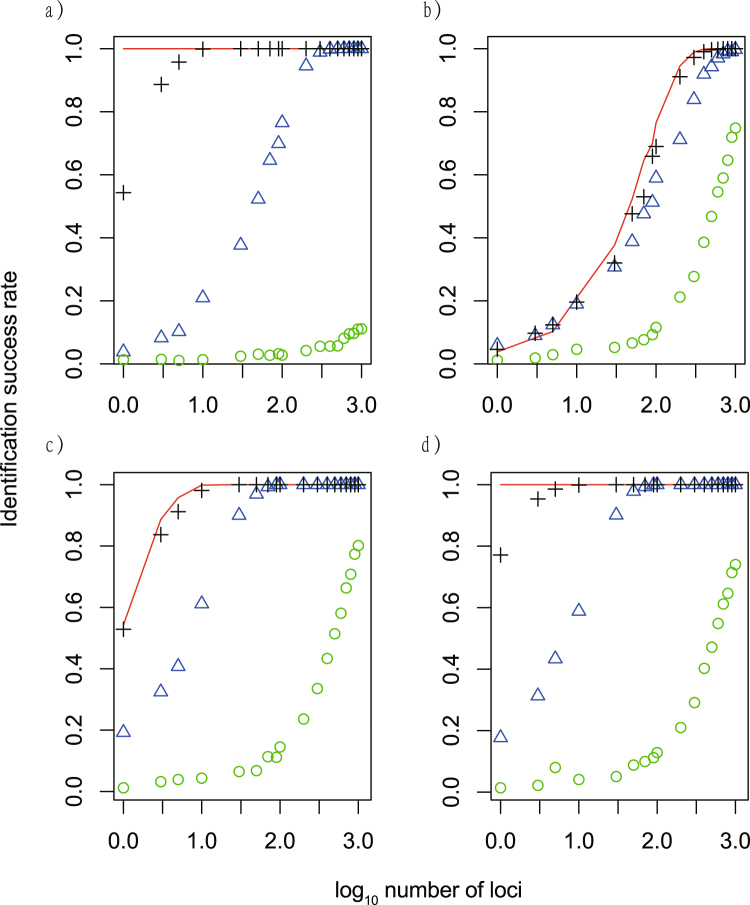
Identification success rate using simulated sequences under different scenarios. (**a**) migration rate equals zero and divergence time equals 700,000 generations (red line), 100,000 generations (black crosses), 10,000 generations (blue triangles), and 1000 generations (green circles); (**b**) divergence time equals 10,000 and migration rate equals 0 (red line), 0.000001 (black crosses), 0.00001 (blue triangles) and 0.0001 (green circles); (**c**) divergence time equals 100,000 and migration rate equals 0 (red line), 0.000001 (black crosses), 0.00001 (blue triangles) and 0.0001 (green circles); (**d**) divergence time equals 700,000 and migration rate equals 0 (red line), 0.000001 (black crosses), 0.00001 (blue triangles) and 0.0001 (green circles).

To explore the effect of divergence time and gene flow on the success rate of species identification, we conducted a series of simulations using twenty thousand loci for two species with a range of splitting times and migration profiles. Five sequences from each species were sampled to calculate species identification success rate. Different number of simulated loci were randomly picked and used to identify species. The identification success rate rose with increasing number of loci included in the analyses in all scenarios (Fig. 4). When there was no migration between the two simulated species, the identification success rate increased with splitting time (Fig. 4a). The simulation with a splitting time of 1,000 generations had the worst identification success rate, only 0.111 even with 1,000 loci used (green circle, Fig. 4a; Supplementary Table S2). The samples with a splitting time of 10,000 generations had low success rates with a small number of loci used, but rose to 1 when more than 400 loci were added to the analyses (blue triangles, Fig. 4a). The samples with a splitting time of 100,000 generations had a success rate of 1 when more than 10 loci were used (black crosses, Fig. 4a). Samples with a splitting time of 700,000 had success rate of 1 for all analyses (red line, Fig. 4a).

When gene flow was considered, high gene flow worked in concert with shallow divergence time to reduce the identification success rate (Fig. 4b-d). A migration rate of 0.0001 (per gene per generation) always led to the worst success rate, and failed to reach a success rate of 1.0 even when all 1,000 loci were used in analyses (green circles, Fig. 4b–d; Supplementary Tables S3–S5). At a migration rate of 0.00001 (blue triangles, Fig. 4b–d) or a migration rate of 0.000001 (black crosses, Fig. 4b–d), the identification success rate improved quickly with increasing number of loci (Fig. 4b–d; Supplementary Tables S3–S5). When the divergence time was greater than 100,000 generations and gene flow was lower than 0.00001, the identification success rate reached 1.0 when more than 90 loci were added to the analysis (Fig. 4c–d; Supplementary Tables S4–S5).

**Figure 5:**
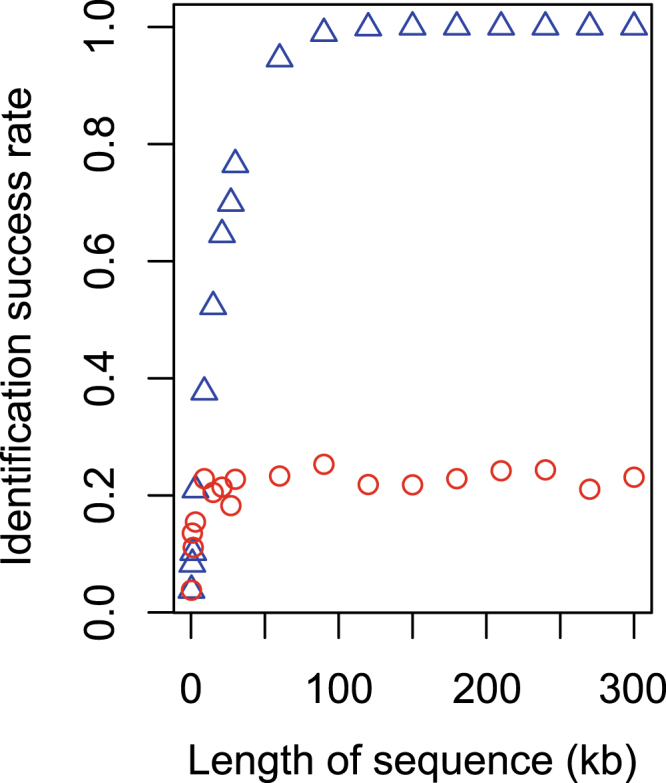
Comparison between success rates of species identification based on a single locus (red circles) and multiple loci (blue triangles). The length of the single locus equals the total length of multiple loci (300 bp each).

To test whether the length of sequence or the number of loci was the key for success in species identification, we simulated a single locus with increasing size matching the total length of multiple loci. We found that increasing the length of a single locus from 300 bp to 9,000 bp improved the success rate slightly, but the success rate did not change when longer sequences were used (Fig. 5 red circles; Supplementary Table S6). In contrast, concatenating more independent loci with the same total length as the single locus continuously improved the identification success rate, until it reached one (Fig. 5 blue triangles; Supplementary Table S6).

### Multilocus DNA barcoding using empirical data

Based on the results from p-distance and species identification analyses of simulated and empirical data, we decided to pick 500 loci for multilocus DNA barcoding. First, we filtered the 4,434 markers developed for all ray-finned fishes and kept 750 loci with the lowest number of missing taxa. Next, we sorted the 750 loci by their average p-distance and picked from them 500 independent loci with large p-distances. This design was implemented both to minimize missing data when applying to ray-finned fishes and to ensure that loci would be variable enough for multilocus DNA barcoding. Information describing the 500 loci is listed in Supplementary Table S7.

Three individuals, 839_3 (*Sini*. *kneri*), 839_6 (*Sini*. *kneri*), and 938_1 (*Sini*. *chuatsi*) were randomly selected. Each of the randomly picked individuals was used to simulate “a putatively unknown” query for identification. Firstly, the p-distance between the unknown query and the other sinipercids in the database was calculated (Supplementary Table S8). Secondly, based on the sorted list of p-distances, we selected five closely related taxa, including the query. For example, for 839_3, we used sequence data of 839_3, *Sini*. *kneri*, *Sini*. *chuatsi*, *Sini*. *undulata* and *Sini*. *obscura* to reconstruct a species tree, in which 839_3 was found to be sister to *Sini*. *kneri* (Supplementary Fig. S2). We then ran a BFD* test to delimitate the unknown query (839_3) with *Sini*. *kneri* using *Sini*. *chuatsi* as outgroup. The BFD* analyses correctly grouped 839_3 (*Sini*. *kneri*) with *Sini*. *kneri* (Table 1). The two other randomly picked samples, 839_6 (*Sini*. *kneri*), and 938_1 (*Sini*. *chuatsi*) were also correctly identified (Supplementary Tables S8 and S9).

**Table 1:** Results for species delimitation on unknown sample 893_3 (*Sini*. *kneri*) using BFD* based on all 500 nuclear loci, missing 20%, 30% and 50% of the 500 loci or missing conspecific of *Sini*. *kneri* in the database.

Data treatment	Model	Marginal likelihood	2lnBF
Using all data	Lumping 839_3 and *Sini*. *kneri*	−1575.80	20.62
	Splitting 839_3 and *Sini*. *kneri*	−1586.11	
Excluding conspecifics of 839_3	Lumping 839_3 and *Sini*. *chuatsi*	−2350.77	
	Splitting 839_3 and *Sini*. *chuatsi*	−1222.90	2255.7
Excluding 20% loci of the 839_3	Lumping 839_3 and *Sini*. *kneri*	−1467.41	26.54
	Splitting 839_3 and *Sini*. *kneri*	−1480.68	
Excluding 30% loci of the 839_3	Lumping 839_3 and *Sini*. *kneri*	−1247.44	22.12
	Splitting 839_3 and *Sini*. *kneri*	−1258.50	
Excluding 50% loci of the 839_3	Lumping 839_3 and *Sini*. *kneri*	−914.60	22.40
	Splitting 839_3 and *Sini*. *kneri*	−925.80	

DNA barcoding using only COI data was unsuccessful. In many cases, the closest taxa of the unknow samples were not their conspecifics either in the tree or measured by p-distances (Supplementary Fig. S3 and Table S10).

### Effect of missing data on multilocus DNA barcoding

When all conspecifics were excluded from the database, the unknown query, 893_3 (*Sini*. *kneri*) was found to be closely related to its sister species *Sini*. *chuatsi* (Supplementary Fig. S4). The p-distances also indicated that the unknown was related to *Sini*. *chuatsi* (Supplementary Table S11). A species delimitation analysis was run with the BFD* method to test whether the unknown should be assigned to *Sini*. *chuatsi* or not. The result of BFD* strongly support the unknown query as a separate species (2lnBF = 2,255.7; Table 1). In other tests, we keep the database intact, but excluded 20%, 30% and 50% of the loci from the unknown query (893_3 *Sini*. *kneri*). We still identified the unknown correctly using the multilocus DNA barcoding approach (Table 1).

## Discussion

Our results demonstrated that the difference between species become more distinct when more independent loci are used. The intra- (red) and interspecific (blue) p-distance between individuals of *Sini*. *chuatsi* and *Sini*. *kneri* were largely overlapping when only COI gene or a few randomly picked nuclear gene were used to calculate the p-distance (Fig. 1). When more loci were added to the analyses, the intra- and interspecific distance became better separated. At 90 loci, a “barcoding gap” between the intra- and interspecific distance emerged. The variance of the intra- and interspecific distances also decreased as the number of loci used in the analyses increased. Based on these findings we conclude that the lack of an apparent barcoding gap between *Sini*. *chuatsi* and *Sini*. *kneri* using COI or a few nuclear genes is due to sampling error. Using more independent loci would likely improve the estimates of population parameters^46^. Similarly, more indepenent loci should improve precision of both the estimated intra- and interspecific genetic distance, resulting in increased discriminatory power (Fig. 3). The same patterns were obeserved in all of our simulated analyses, namely that the species identification success rate rose with increasing number of loci (Fig. 4). Interestingly, using longer genes instead of more genes did not improve species identification (Fig. 5).

Gene flow between sister species can cause problems that are similar to those caused by a lack of divergence. *Sini*. *chuatsi* and *Sini*. *kneri* were estimated split at around 800 thousand generations ago, with uni-directional introgression flowing from *Sini*. *kneri* to *Sini*. *chuatsi*, m_1>0_ = 0.640. Therefore, the lack of reciprocal monophyly or barcoding gap between *Sini*. *chuatsi* and *Sini*. *kneri* using COI or a few nuclear loci could, in fact, be caused by gene flow between the two species rather than the short divergence time originally hypothesized by us.

*Sicydium altum* and *Sicy*. *adelum* were estimated to have split very recently, t_0_ = 0.003195. Bi-directional gene flow was estimated as 0.494 from *Sicy*. *altum* to *Sicy*. *adelum*, and 0.502 from *Sicy*. *adelum* to *Sicy*. *altum*. All of our analyses could not differentiate between *Sicy*. *altum* and *Sicy*. *adelum* genetically. Structure analysis (Supplementary Fig. S1), species identification and p-distance assessments (Figs 2 and 3) all indicated that *Sicy*. *altum* and *Socy*. *adelum* are indistinguishable. Accordingly, we suggest that the taxonomic status of *Sicy*. *altum* and *Sicy*. *adelum* be revisited by a more detailed morphological analysis.

It is difficult to tell whether gene flow or short species divergence time played a more prominent role in obstructing DNA barcoding. It has been reported that a considerable proportion of animal species do not form monophyletic groups^47,48^, but the causes for such patterns have not yet been fully explored. From the results of our empirical and simulated analyses, we conclude that when the splitting time between sister species was more than 100,000 generations old and the migration rate was lower than 0.00001, using multilocus DNA barcoding (with more than 90 loci) we could correctly determine the species status of unknown samples, whereas single-locus DNA barcoding suffered from lacking of power in species discrimination.

A suite of universal gene markers that could be used on a whole group of organisms is a prerequisite for multilocus DNA barcoding. Because of improvements in sequencing technology and the increasing number of publicly accessable genome data bases, more and more genome-scale markers have been developed for different group of organisms, such as turtles^49^, birds^50^, tapeworms^51^, flower flies^52^, plants^53^, echinoderms^54^, insects^55^ and vertebrates^35^. Some of these markers can be applied across broad groups of organisms, whereas other have only been tested for restricted groups. We predict that obtaining suitable sets of markers for multilocus DNA barcoding will not be a limitation, but a lot of testing will need to be carried out across a broad range of taxa before an agreed set of common markers can be established for each major group of organisms.

Our pick of 500 markers for ray-finned fishes has been tested in major lineages of fishes (33 families and 11 orders). We chose markers that were found to be present in most groups of fishes and that were variable across groups. We recommend using them as standard multilocus DNA barcode markers for all ray-finned fishes. Our results indicate that more than 90 loci should be enough for species identification, but we advocate using the complete set of 500 loci, as there is almost no extra cost in capturing 500 rather than 90 loci. Additionally, targeting more loci provides insurance against missing data. We found that missing 20%, 30% and up to 50% loci in the unknown sample had no effect in identification success.

Other alternatives to collecting large datasets for DNA barcoding include genome skimming^56^ and whole-chloroplast genome sequencing^57^. Genome skimming employs low-coverage shotgun sequencing of genomic DNA, which circumvents the need for PCR, avoiding the needs for univerals primers. Because genome skimming is unselective, it involves collecting a lot of data that ultimately is not used, but requires data storage and analysis resources. Low-coverage shotgun sequencing also yields a high proportion of missing data. Sequencing genomes of chloroplasts or other organelles is focused on a single long sequence, which tends to yield low success rate of species identification, as shown in our simulation.

Dowton *et al*.^26^ proposed an pipeline integrating species tree reconstruction and species delimitation. They used Beast* to build a species tree^58^, and took the species tree as the guide tree for delimitating species using BPP^59,60^. Our method is similar to the method of Dowton *et al*.^26^. We first screened the reference database for individuals from closely related speices based on p-distance between the unknow query and sequences in the database. We only choose four closely related species as potential conspecies or sister species. We think the number of species selected is enough for the current study, because our p-distance calculation was based on many independent loci, which reduced random error. The small number of selected species could also help to relieve computational burden associated with reconstructing the species tree in the second step. Using a combination of RAxML and ASTRAL program, we could reconstruct a specis tree of five taxa, four selected species plus the query in minutes using 500 loci. In the last step, we included only three taxa, one conspecific or sister species, one outgroup species and the unknown query for species delimiation using BFD*, which also saved computation time. Our multilocus barcoding approach is conceptually similar to the method of Dowton *et al*.^26^. However, we used many more loci (hundreds vs two), and focused on rooted trees with only three taxa, so it should be more powful and tractable than the method of Dowton *et al*. We anticipate that the computational burden associated with multilocus DNA barcoding will be further reduced as new algorithms are developed, to make multilocus barcoding a real-time tool.

Finally, from a practical standpoint, multilocus barcoding through target gene enrichment is efficient. We estimate around $90 for the total cost of capturing and sequencing 500 loci per sample, which is less than the cost of amplifying and sequencing 10 loci using the tranditional methods of PCR and Sanger sequening. The cost of target gene capture comprises: library prep, $50; RNA baits, $32; and sequencing, $8 per sample. The major costs are associated with the purchase of commercial RNA bait kits and the library preparation step, which can be lowered by purchasing kits in bulk and by using robots to automate library preparation. Finally, we selected 20 loci that have few missing data and long sequence length and recommend these for who want to use regular PCR and Sanger sequencing to collect multilocus data for species identification (Supplementary Table S12). These markers also can be used for phylogenetic study in the ray-finned fishes.

## Materials and Methods

### Taxa sampling, target gene enrichment, sequencing and reads assembly

We used the sequence data of the 4,434 loci of the sinipercids from Song *et al*.^61^. The samples included five *Coreoperca whiteheadi*, one *Sini*. *scherzeri*, five *Sini*. *obscura*, two *Sini*. *undulata*, three *Sini*. *roulei*, five *Sini*. *chuatsi* and five *Sini*. *kneri*.

For the goby study, nine *Sicy*. *altum* and seven *Sicy*. *adelum* were collected from Costa Rica. Total genomic DNA was extracted from fin clips using a Tissue DNA kit (Omega Bio-tek, Norcross, GA, USA) and the concentration of DNA was quantified using NanoDrop 3300 Fluorospectrometer (Thermo Fisher Scientific, Wilmington, DE, USA). The goby samples were enriched and sequenced for the same 4,434 loci. The amount of DNA used for library preparation was 1 μg for each sample. The DNA sample was first sheared to 250 bp using a Covaris M220 Focused-ultrasonicator^TM^ (Covaris, Inc. Massachusetts, USA). A MYbaits kit containing baits for the 4,434 loci was synthesized at MYcroarray (Ann Arbor, Michigan, USA). The baits were designed on sequences of *Oreochromis niloticus* with 3 × tiling. Blunt-end repair, adapter ligation, fill-in, pre-hybridization PCR and target gene enrichment steps followed the protocol of cross-species gene capture^35^. The enriched libraries were amplified with indexed primers, pooled equimolarly and sequenced on a lane of Illumina HiSeq. 2500 platform with other samples. The raw reads were parsed to separate file for each species according to the indices on the adapter. Reads assembling followed the pipeline of Yuan *et al*.^51^. Mitochondrial COI gene of both the sinipercids and the gobies was also amplified and sequenced using Sanger sequencing to compare COI barcoding with multilocus DNA barcoding using two pairs of primers (siniF: AACCAGCGAGCATCCATCTA and siniR: CAGTGGACGAAAGCAGCAAC for the sinipercids; sicyF: GGTTGTGTTGAGGTTTCGGT and sicyR: TCCGAGCCGAACTAAGTCAA for *Sicydium*).

### Effect of increasing number of loci on species discrimination

Our assumption was that individuals of recently diverged species should be more discernible using many loci than using fewer loci. Thus, we calculated p-distance among 10 individuals of *Siniperca*, including five *Sini*. *chuatsi* and five *Sini*. *kneri*, using different number of loci to test this hypothesis. Loci with no missing data in all 10 individuals of *Siniperca* were picked using a custom Perl scripts (picktaxagene.pl). The obtained 2,612 loci were then sorted by their average p-distance (distoutlier.pl), so outlier loci with extreme large p-distance could be checked by eye to spot bad data or bad alignment. After removing the bad data, a different number (1, 3, 10, 30, 50, 70, 90, 100, 200, 300, 400, 500, 600, 700, 800, 900, 1000, 2000) of loci were randomly picked and concatenated (samplegene.pl) for calculating p-distance among individuals (gapdis.pl). The sampling at each level of different number of loci was repeated two hundred times. The p-distance among individuals vs. the number of loci used was drawn with GraphPad Prism 5 (San Diego, California). To check the effect of increasing number of loci on species discrimination, the “all species barcodes” criteria was applied, that is queries was considered successfully identified when they were followed by all conspecifics according to their barcode^44^. Custom Perl script was used to calculate the rate of successful identification for 200 replicates at each level of number of loci used (ID_correct_rate.pl). Among individual p-distance and rate of successful identification also were calculated for the *Sicydium*. Sequences of COI gene also were used to calculate p-distance between individuals from the same species and from different species to compare with the results of nuclear genes. Spider^28^ was used to optimize barcoding distance threshold and to identify species using COI sequences as suggested by Collins and Cruickshank^27^. The final number of loci recommended for DNA barcoding was chosen based on the effect of increasing number of loci on the success rate of species discrimination.

### Estimating species divergence and gene flow in the empirical data

Gene flow and differentiation time of *Sini*. *chuatsi* and *Sini*. *kneri* was estimated using IMa2 program with 200 loci^62^. The MCMC was run for 10 million generations with sample recorded every hundred generations. The number of chains was set to 20. The running parameters were set as -q2, -m1, -t3, -b 10000000, d100, -hn20 and -s123. An additional run was performed with the same parameter but different seeds –s111. These two run showed decent mixing, and similar results, so we combined results from the two runs. Similar runs were done for the two species of *Sicydium*. The genetic differentiation between the two species of *Siniperca* and the two species of *Sicydium* also was estimated using Structure 2.3.4^63^. Three iterations for 100,000 generations (using a 100,000 burnin) were run for each value of K (number of population clusters) ranging from 1 to 3. To identify the number of population clusters that captures the major structure in the data, Structure Harvester^64^ was used to calculate the peak value for delta K^65^.

### Simulating sister species sequence data with different divergence times and gene flow

We simulated two diverging species with various splitting time and migration rates to explore the effect of changing these two factors on species discrimination over a broader range of parameter space. According to the IMa2 results of the empirical data, the splitting time was set as 1,000, 10,000, 100,000, and 700,000 generations. The migration rate was set as 0, 0.000001, 0.00001, and 0.0001 per generation. The simulation with 1,000 generations splitting time was combined with only 0 migration rate, because the two simulated species were already indistinguishable under 1,000 generations splitting time even when there was no gene flow in the simulation. The simulations with 10,000, 100,000, and 700,000 generation splitting time were combined with all four migration rates. Fastsimcoal2^66,67^ was used to generate the simulated data. Twenty thousand replicates were simulated for each scenario. The effective population size used for simulation was 20,000 in the ancestor species and the two descendant species. The mutation rate was set to 2 × 10^−8^. Five sequences were sampled from each simulated species. The simulated data were used to calculate p-distance among individuals of the same and different species. Species identification success rate applying “all species barcodes” criteria was calculated as described above. Identification success rate using different number (1, 3, 5, 10, 30, 50, 70, 90, 100, 200, 300, 400, 500, 600, 700, 800, 900, 1000) of simulated loci was plotted against species splitting time and migration rate using R^68^.

### A three-step multilocus DNA barcoding pipeline

**Figure 6:**
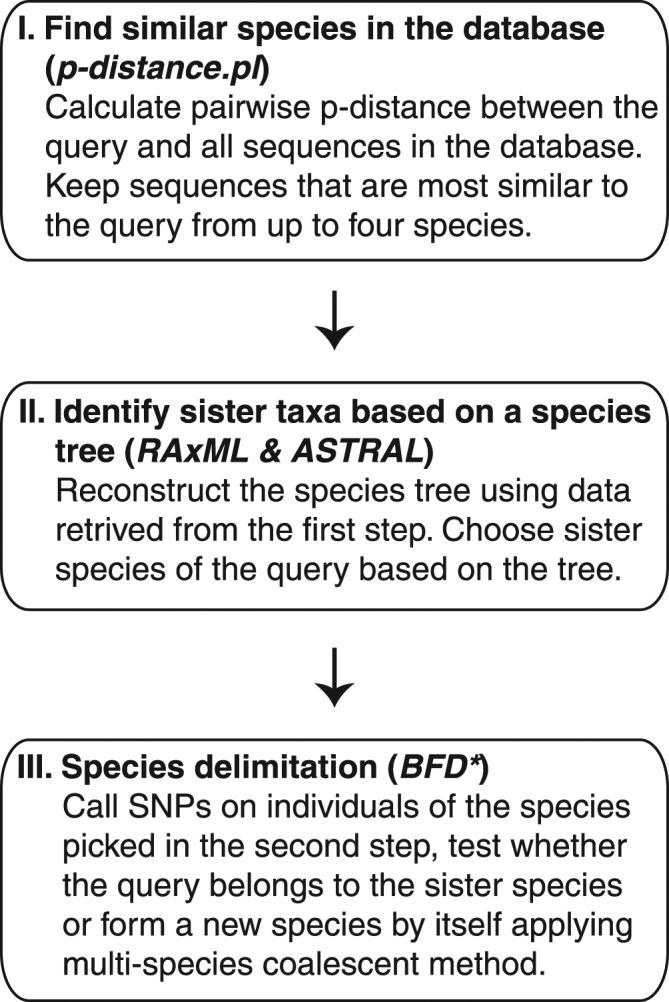
A three-step multilocus DNA barcoding pipeline.

It is straightforward to use distance based methods to reveal divergence of two sister species in the empirical and simulated data. But for more than two species, distance based species identification becomes more complicated. Firstly, an arbitrary barcoding threshold is needed to judge whether the query is one of the species represented in the database or is a new and distinct species. Secondly, the shortest distance does not necessarily guarantee a sister species relationship, because sister species with long branches might be less similar to the query species than a non-sister species with a short branch. To avoid these risks, we propose a three-step DNA barcoding method (Fig. 6).

In the first step, p-distances between the query and all sequences in the database are calculated. The sequences that are similar to the query are kept for subsequent analyses (p-distance.pl). This is a fast screening process to retrieve all sequences from potential conspecifics or sister species. Because the closest sequence might not be from a conspecifics or sister species, sequences from up to four species are kept. In the second step, a species tree is reconstructed using the sequences from the first step to identify potential conspecifics or sister species of the query using RAxML version 8^69^ and ASTRAL 4.10.6^70–72^. Individual gene trees are inferred using RAxML with the GTRGAMMA model, and then a species tree is recovered from those gene trees using ASTRAL. The potential conspecifics or sister species to the query are then chosen based on the phylogenetic relationship depictured in the species tree. In the third step, species delimitation is done using a Bayes factor delimitation approach, BFD*^73^. Single nucleotides polymorphism (SNP) data are retrieved from the sequencing reads of the species chosen in step two and used for the BFD* analysis. A path sampling with 48 steps was conducted to estimate the marginal likelihood with a Markov chain Monte Carlo (MCMC) chain length of 200,000 and a pre-burnin of 50,000 following the recommended settings in BFD*^73^. The strength of support for compared hypotheses was evaluated from Bayes factor scale, 2ln(BF) using the framework of Kass and Raftery^74^. The BF scale is as follows: 0 < 2ln(BF) < 2 is not worth more than a bare mention, 2 < 2ln(BF) > 6 means positive evidence, 6 < 2ln(BF) < 10 represents strong support, and 2ln(BF) > 10 represents decisive support. If the result of BFD* analysis does not support two separate species, the query will be assigned to the “sister species”; otherwise, the query will be considered as a new species with its sequences add to the database and further study on its species status will be recommended.

The final set of selected markers was used for testing the above-described three-step multilocus DNA barcoding in the sinipercids, including 26 individuals of seven species. An individual of *Sini*. *kneri* or *Sini*. *chuatsi* was randomly chosen as unknown query that needs to be identified. The sequences of the unknown specimens and all other sequences in the database were aligned using Clustal Omega v1.1.1^75^. Custom Perl scripts, concatnexus.pl and gapdis.pl were used to concatenate the sequences of individual loci, to calculate their p-distance between the query and the sample in the database, and sorted them by the p-distance to find all individuals that are close to the query sample.

### Testing effect of missing data in the database or in the query on the success rate of species identification

To test if our method could identify new species when the sequences of conspecifics are not in the database, all samples of *Sini*. *kneri* were removed from the database except that one random selected *Sini*. *kneri* individual was left as query. To access the effect of missing data in the query sample, one *Sini*. *kneri* was selected as an unknown sample, and 20 percent, 30 percent, and 50 percent of its loci were excluded, then the data were used for multilocus DNA barcoding analysis.

### Data availability

The raw sequence reads are available in NCBI repository (accession: PRJNA373944 and PRJNA355377). The sequences alignments and Perl scripts can be found in Supplementary Information.

## Electronic supplementary material

Supplementary Information

Perl scripts

Dataset 1

Dataset 2

Dataset 3

Dataset 4
