# Supplementary material for: Multilocus DNA barcoding – Species Identification with Multilocus Data
Source: Sci Rep. 2017 Nov 30;7:16601. doi: 10.1038/s41598-017-16920-2 (PMC5709489; doi:10.1038/s41598-017-16920-2)
Supplement: Supplementary file 1 — Supplementary Information [file 41598_2017_16920_MOESM1_ESM.pdf]

Multilocus DNA barcoding – Species Identification with Multilocus Data

Junning Liu, Jiamei Jiang, Shuli Song, Luke Tornabene, Ryan Chabarria, Gavin J P Naylor, Chenhong Li

Supplementary Table S1 – S12

Supplementary Fig. S1 – S4

Supplementary Table S1. Success rate of identification in the samples of *Siniperca* increased with more loci added in the analyses

Number of loci	Identification success rate
1	0.412
3	0.7145
5	0.8475
10	0.9685
30	0.988
50	0.9915
70	0.998
90	1
100	1
200	1
300	1
400	1
500	1
600	1
700	1
800	1
900	1
1000	1
2000	1

Supplementary Table S2. Success rate of identification in simulated data increased with splitting time

Number of loci	Splitting time (generations)			
	1000	10000	100000	700000
1	0.0125	0.038	0.543	1
3	0.014	0.0825	0.8865	1
5	0.0105	0.1025	0.9575	1
10	0.013	0.2095	0.9985	1
30	0.024	0.3765	1	1
50	0.0305	0.5225	1	1
70	0.027	0.6455	1	1
90	0.032	0.699	1	1
100	0.0275	0.765	1	1
200	0.042	0.9455	1	1
300	0.0555	0.989	1	1
400	0.056	0.9985	1	1
500	0.057	1	1	1
600	0.081	1	1	1
700	0.095	1	1	1
800	0.0965	1	1	1
900	0.11	1	1	1
1000	0.111	1	1	1

Supplementary Table S3. Success rate of identification in simulated data with different migration rate (splitting time equals 10000)

Number of loci	Migration rate			
	0	0.000001	0.00001	0.0001
1	0.038	0.0465	0.0585	0.0117
3	0.0825	0.097	0.0895	0.0185
5	0.1025	0.1235	0.124	0.029
10	0.2095	0.196	0.1895	0.046
30	0.3765	0.32	0.307	0.052
50	0.5225	0.476	0.3875	0.066
70	0.6455	0.53	0.476	0.0765
90	0.699	0.6585	0.5125	0.0925
100	0.765	0.6895	0.5895	0.1155
200	0.9455	0.911	0.7115	0.212
300	0.989	0.972	0.8385	0.277
400	0.9985	0.9905	0.919	0.3855
500	1	0.9965	0.9425	0.4675
600	1	0.998	0.9705	0.5455
700	1	1	0.9835	0.5896
800	1	1	0.9925	0.646
900	1	1	0.99	0.719
1000	1	1	0.9975	0.748

Supplementary Table S4. Success rate of identification in simulated data with different migration rate (splitting time equals 100000)

Number of loci	Migration rate			
	0	0.000001	0.00001	0.0001
1	0.534	0.5282	0.1925	0.012
3	0.8865	0.837	0.3245	0.032
5	0.9575	0.912	0.4075	0.039
10	0.9985	0.981	0.6115	0.0435
30	1	1	0.9	0.065
50	1	1	0.9691	0.068
70	1	1	0.981	0.1135
90	1	1	1	0.1115
100	1	1	1	0.145
200	1	1	1	0.236
300	1	1	1	0.3355
400	1	1	1	0.4335
500	1	1	1	0.514
600	1	1	1	0.581
700	1	1	1	0.6635
800	1	1	1	0.708
900	1	1	1	0.7735
1000	1	1	1	0.8015

Supplementary Table S5. Success rate of identification in simulated data with different migration rate (splitting time equals 700000)

Number of loci	Migration rate			
	0	0.000001	0.00001	0.0001
1	1	0.771	0.177	0.014
3	1	0.9535	0.313	0.022
5	1	0.9855	0.4335	0.08
10	1	0.9985	0.5885	0.041
30	1	1	0.901	0.0505
50	1	1	0.9785	0.088
70	1	1	0.9935	0.0995
90	1	1	0.9995	0.112
100	1	1	1	0.1285
200	1	1	1	0.21
300	1	1	1	0.291
400	1	1	1	0.4025
500	1	1	1	0.4715
600	1	1	1	0.5485
700	1	1	1	0.612
800	1	1	1	0.6465
900	1	1	1	0.714
1000	1	1	1	0.74

Supplementary Table S6. Success rate of identification based on a single locus with increasing length or based on multiple loci (300 bp for each locus) with the same total length as the single locus

Sequence length (bp)	Identification success rate	
	Single locus	Multiple loci
300	0.038	0.038
900	0.1355	0.0825
1500	0.111	0.1025
3000	0.1545	0.2095
9000	0.2285	0.3765
15000	0.205	0.5225
21000	0.214	0.6455
27000	0.1825	0.699
30000	0.2275	0.765
60000	0.233	0.9455
90000	0.253	0.989
120000	0.2185	0.9985
150000	0.218	1
180000	0.2285	1
210000	0.242	1
240000	0.2435	1
270000	0.2105	1
300000	0.231	1

Supplementary Table S7. Name, chromosomal position and description of the 500 markers used for multilocus DNA barcoding

Organisms	Chromosome /Scaffolds	Start Position	Ending Position	Ensembl code	Short name	Strand	Annotation
Danio_rerio	1	18296121	18296321	ENSDARG00000017744	smc2	1	structural maintenance of chromosomes 2 [Source:ZFIN;Acc:ZDB-GENE-030131-105]
Danio_rerio	1	27017867	27018101	ENSDARG00000031632	chst10	1	carbohydrate sulfotransferase 10 [Source:ZFIN;Acc:ZDB-GENE-040808-40]
Danio_rerio	1	28199081	28201810	ENSDARG00000060620	lig4	1	ligase IV, DNA, ATP-dependent [Source:ZFIN;Acc:ZDB- GENE-070820-10]
Danio_rerio	1	42238686	42238912	ENSDARG00000036967	smox	1	spermine oxidase [Source:ZFIN;Acc:ZDB-GENE-031201- 3]
Danio_rerio	1	46310620	46310764	ENSDARG00000008279	map2k7	1	mitogen-activated protein kinase kinase 7 [Source:ZFIN;Acc:ZDB-GENE-090312-186]
Danio_rerio	1	53848151	53848280	ENSDARG00000059900	tbc1d9	1	TBC1 domain family, member 9 (with GRAM domain) [Source:ZFIN;Acc:ZDB-GENE-060810-33]
Danio_rerio	2	13632602	13633206	ENSDARG00000054984	ZBTB41	1	zinc finger and BTB domain containing 41 [Source:HGNC Symbol;Acc:24819]
Danio_rerio	2	2479674	2479521	ENSDARG00000074571	GPAA1	-1	glycosylphosphatidylinositol anchor attachment protein 1 homolog (yeast) [Source:HGNC Symbol;Acc:4446]
Danio_rerio	2	27975885	27975672	ENSDARG00000015564	dhcr7	-1	7-dehydrocholesterol reductase [Source:ZFIN;Acc:ZDB- GENE-030912-9]
Danio_rerio	2	32911649	32911782	ENSDARG00000060929	rnf220a	1	ring finger protein 220a [Source:ZFIN;Acc:ZDB-GENE- 060929-1006]
Danio_rerio	2	35781500	35781650	ENSDARG00000060767	smg7	1	Smg-7 homolog, nonsense mediated mRNA decay factor (C. elegans) [Source:ZFIN;Acc:ZDB-GENE-030131-9122]
Danio_rerio	2	3899585	3899750	ENSDARG00000058995	JMJD4	1	jumonji domain containing 4 [Source:HGNC Symbol;Acc:25724]
Danio_rerio	3	26120377	26120509	ENSDARG00000038094	clec16a	1	C-type lectin domain family 16, member A [Source:ZFIN;Acc:ZDB-GENE-040426-951]
Danio_rerio	3	32021560	32021284	ENSDARG00000037928	ddx42	-1	DEAD (Asp-Glu-Ala-Asp) box polypeptide 42 [Source:ZFIN;Acc:ZDB-GENE-050706-53]

Organisms	Chromosome /Scaffolds	Start Position	Ending Position	Ensembl code	Short name	Strand	Annotation
Danio_rerio	3	34020247	34020039	ENSDARG00000032129	gtf2f1	-1	general transcription factor IIF, polypeptide 1 [Source:ZFIN;Acc:ZDB-GENE-030131-4557]
Danio_rerio	3	35707286	35707503	ENSDARG00000060207	traf7	1	TNF receptor-associated factor 7 [Source:ZFIN;Acc:ZDB-GENE-070112-2212]
Danio_rerio	3	53980618	53980780	ENSDARG00000007092	xab2	1	XPA binding protein 2 [Source:ZFIN;Acc:ZDB-GENE- 040426-685]
Danio_rerio	4	15053537	15053670	ENSDARG00000034823	copg2	1	coatomer protein complex, subunit gamma 2 [Source:ZFIN;Acc:ZDB-GENE-000208-8]
Danio_rerio	4	19418591	19417945	ENSDARG00000009252	napepld	-1	N-acyl phosphatidylethanolamine phospholipase D [Source:ZFIN;Acc:ZDB-GENE-030131-3856]
Danio_rerio	4	730788	730651	ENSDARG00000063686	XRCC6BP1	-1	XRCC6 binding protein 1 [Source:HGNC Symbol;Acc:29452]
Danio_rerio	5	16234556	16233655	ENSDARG00000079611	SEMA4C	-1	sema domain, immunoglobulin domain (Ig), transmembrane domain (TM) and short cytoplasmic domain, (semaphorin) 4C [Source:HGNC Symbol;Acc:10731]
Danio_rerio	5	17719179	17718607	ENSDARG00000035622	xbp1	-1	X-box binding protein 1 [Source:ZFIN;Acc:ZDB-GENE- 011210-2]
Danio_rerio	5	17933857	17933701	ENSDARG00000062579	kremen1	-1	kringle containing transmembrane protein 1 [Source:ZFIN;Acc:ZDB-GENE-070705-262]
Danio_rerio	5	19529890	19531189	ENSDARG00000058232	fbrsl1	1	fibrosin-like 1 [Source:ZFIN;Acc:ZDB-GENE-070705- 487]
Danio_rerio	5	20715305	20715438	ENSDARG00000035608	pgam5	1	phosphoglycerate mutase family member 5 [Source:ZFIN;Acc:ZDB-GENE-030131-683]
Danio_rerio	5	26211472	26210808	ENSDARG00000002830	trmt2a	-1	trm2 tRNA methyltransferase 2 homolog A (S. cerevisiae) [Source:ZFIN;Acc:ZDB-GENE-030131-6876]
Danio_rerio	5	28570695	28570908	ENSDARG00000035515	TM2D2	1	TM2 domain containing 2 [Source:HGNC Symbol;Acc:24127]
Danio_rerio	5	32228605	32228818	ENSDARG00000061061	dpagt1	1	dolichyl-phosphate (UDP-N-acetylglucosamine) N-acetylglucosaminephosphotransferase 1 (GlcNAc-1-P transferase) [Source:ZFIN;Acc:ZDB-GENE-060526-18]
Danio_rerio	5	32647796	32647945	ENSDARG00000061013	ankfy1	1	ankyrin repeat and FYVE domain containing 1 [Source:ZFIN;Acc:ZDB-GENE-041222-1]

Organisms	Chromosome /Scaffolds	Start Position	Ending Position	Ensembl code	Short name	Strand	Annotation
Danio_rerio	5	35254252	35254827	ENSDARG00000060374	ttll11	1	tubulin tyrosine ligase-like family, member 11 [Source:ZFIN;Acc:ZDB-GENE-061013-747]
Danio_rerio	5	38170455	38170229	ENSDARG00000035330	taf1	-1	TAF1 RNA polymerase II, TATA box binding protein (TBP)-associated factor [Source:ZFIN;Acc:ZDB-GENE-030131- 5576]
Danio_rerio	5	67591469	67590842	ENSDARG00000078655	inpp5e	-1	inositol polyphosphate-5-phosphatase [Source:ZFIN;Acc:ZDB-GENE-050809-23]
Danio_rerio	5	71737004	71737156	ENSDARG00000041908	usp39	1	ubiquitin specific peptidase 39 [Source:ZFIN;Acc:ZDB-GENE-030131-966]
Danio_rerio	6	16563331	16563184	ENSDARG00000079480	UNC80	-1	unc-80 homolog (C. elegans) [Source:HGNC Symbol;Acc:26582]
Danio_rerio	6	2271212	2271364	ENSDARG00000077753	CABZ01069216.1	1	Uncharacterized protein [Source:UniProtKB/TrEMBL;Acc:F1Q941]
Danio_rerio	6	23710138	23711977	ENSDARG00000052351	ZXDA	1	zinc finger, X-linked, duplicated A [Source:HGNC Symbol;Acc:13198]
Danio_rerio	6	27062551	27062696	ENSDARG00000076094	KLHL30	1	kelch-like 30 (Drosophila) [Source:HGNC Symbol;Acc:24770]
Danio_rerio	6	29862704	29860991	ENSDARG00000079670	LRRC7	-1	leucine rich repeat containing 7 [Source:HGNC Symbol;Acc:18531]
Danio_rerio	6	40759925	40759718	ENSDARG00000004696	eefsec	-1	eukaryotic elongation factor, selenocysteine-tRNA- specific [Source:ZFIN;Acc:ZDB-GENE-051120-72]
Danio_rerio	6	46387908	46388030	ENSDARG00000044182	stau1	1	staufen, RNA binding protein, homolog 1 (Drosophila) [Source:ZFIN;Acc:ZDB-GENE-030131-6372]
Danio_rerio	6	49674955	49675086	ENSDARG00000003307	stx16	1	syntaxin 16 [Source:ZFIN;Acc:ZDB-GENE-060810-113]
Danio_rerio	6	51721311	51721048	ENSDARG00000052073	blcap	-1	bladder cancer associated protein [Source:ZFIN;Acc:ZDB-GENE-000330-7]
Danio_rerio	6	59641680	59641819	ENSDARG00000045019	aamp	1	angio-associated, migratory cell protein [Source:ZFIN;Acc:ZDB-GENE-040426-2370]
Danio_rerio	7	19207491	19207711	ENSDARG00000079684	taf6l	1	TAF6-like RNA polymerase II, p300/CBP-associated factor (PCAF)-associated factor [Source:ZFIN;Acc:ZDB-GENE- 030131-6789]
Danio_rerio	7	25640133	25639647	ENSDARG00000077256	nat8l	-1	N-acetyltransferase 8-like [Source:ZFIN;Acc:ZDB- GENE-030729-4]
Danio_rerio	7	25671744	25671928	ENSDARG00000061411	whsc2	1	Wolf-Hirschhorn syndrome candidate 2 [Source:ZFIN;Acc:ZDB-GENE-030131-6400]

Organisms	Chromosome /Scaffolds	Start Position	Ending Position	Ensembl code	Short name	Strand	Annotation
Danio_rerio	7	35705170	35704861	ENSDARG00000003495	madd	-1	MAP-kinase activating death domain [Source:ZFIN;Acc:ZDB-GENE-030131-8076]
Danio_rerio	7	69813677	69813832	ENSDARG00000053946	dhx38	1	DEAH (Asp-Glu-Ala-His) box polypeptide 38 [Source:ZFIN;Acc:ZDB-GENE-040426-1144]
Danio_rerio	7	72271760	72271906	ENSDARG00000073741	RAP1GDS1	1	RAP1, GTP-GDP dissociation stimulator 1 [Source:HGNC Symbol;Acc:9859]
Danio_rerio	8	12264492	12264303	ENSDARG00000063005	anapc7	-1	anaphase promoting complex subunit 7 [Source:ZFIN;Acc:ZDB-GENE-081104-113]
Danio_rerio	8	15495730	15497450	ENSDARG00000013841	abl2	1	v-abl Abelson murine leukemia viral oncogene homolog 2 [Source:ZFIN;Acc:ZDB-GENE-020809-2]
Danio_rerio	8	16150702	16150405	ENSDARG00000096480	AGBL4=(2=of=2)	-1	ATP/GTP binding protein-like 4 [Source:HGNC Symbol;Acc:25892]
Danio_rerio	8	16833218	16833082	ENSDARG00000022418	faf1	-1	Fas associated factor 1 [Source:ZFIN;Acc:ZDB-GENE- 040426-2863]
Danio_rerio	8	26746458	26747314	ENSDARG00000055763	tmem115	1	transmembrane protein 115 [Source:ZFIN;Acc:ZDB-GENE- 050913-157]
Danio_rerio	8	48846754	48846307	ENSDARG00000059988	gpr153	-1	G protein-coupled receptor 153 [Source:ZFIN;Acc:ZDB- GENE-050823-7]
Danio_rerio	8	53597345	53597189	ENSDARG00000029556	kansl3	-1	KAT8 regulatory NSL complex subunit 3 [Source:ZFIN;Acc:ZDB-GENE-050809-132]
Danio_rerio	8	55543644	55544041	ENSDARG00000075162	CHDH	1	choline dehydrogenase [Source:HGNC Symbol;Acc:24288]
Danio_rerio	8	7503582	7503275	ENSDARG00000022974	wdr13	-1	WD repeat domain 13 [Source:ZFIN;Acc:ZDB-GENE- 050522-274]
Danio_rerio	9	17940234	17942085	ENSDARG00000078001	kbtbd7	1	kelch repeat and BTB (POZ) domain containing 7 [Source:ZFIN;Acc:ZDB-GENE-070912-204]
Danio_rerio	9	22386580	22386710	ENSDARG00000010281	xpo4	1	exportin 4 [Source:ZFIN;Acc:ZDB-GENE-030131-3062]
Danio_rerio	9	31672114	31672344	ENSDARG00000075156	TBC1D4	1	TBC1 domain family, member 4 [Source:HGNC Symbol;Acc:19165]
Danio_rerio	9	32772715	32772533	ENSDARG00000001835	nalcn	-1	sodium leak channel, non-selective [Source:ZFIN;Acc:ZDB-GENE-050410-12]
Danio_rerio	9	33330878	33330543	ENSDARG00000056160	hspd1	-1	heat shock 60kD protein 1 (chaperonin) [Source:ZFIN;Acc:ZDB-GENE-021206-1]

Organisms	Chromosome /Scaffolds	Start Position	Ending Position	Ensembl code	Short name	Strand	Annotation
Danio_rerio	9	3384683	3384995	ENSDARG00000013125	dlx1a	1	distal-less homeobox gene 1a [Source:ZFIN;Acc:ZDB- GENE-990415-48]
Danio_rerio	9	34392307	34393691	ENSDARG00000061791	nyx	1	nyctalopin [Source:ZFIN;Acc:ZDB-GENE-061026-3]
Danio_rerio	9	35088975	35088207	ENSDARG00000000001	slc35a5	-1	solute carrier family 35, member A5 [Source:ZFIN;Acc:ZDB-GENE-030616-55]
Danio_rerio	9	39400711	39400872	ENSDARG00000030789	ddx18	1	DEAD (Asp-Glu-Ala-Asp) box polypeptide 18 [Source:ZFIN;Acc:ZDB-GENE-030131-9685]
Danio_rerio	9	52779281	52778432	ENSDARG00000004712	tbr1b	-1	T-box, brain, 1b [Source:ZFIN;Acc:ZDB-GENE-000323- 1]
Danio_rerio	9	5939584	5939378	ENSDARG00000077983	GPR89B	-1	G protein-coupled receptor 89B [Source:HGNC Symbol;Acc:13840]
Danio_rerio	9	6336765	6336945	ENSDARG00000056102	uxs1	1	UDP-glucuronic acid decarboxylase 1 [Source:ZFIN;Acc:ZDB-GENE-020419-37]
Danio_rerio	10	11300795	11300598	ENSDARG00000063194	lin54	-1	lin-54 homolog [Source:ZFIN;Acc:ZDB-GENE-060929- 440]
Danio_rerio	10	11740007	11740197	ENSDARG00000063161	ppwd1	1	peptidylprolyl isomerase domain and WD repeat containing 1 [Source:ZFIN;Acc:ZDB-GENE-070615-16]
Danio_rerio	10	17047692	17048148	ENSDARG00000078056	RNF208	1	ring finger protein 208 [Source:HGNC Symbol;Acc:25420]
Danio_rerio	10	27820654	27819424	ENSDARG00000056427	auts2	-1	autism susceptibility candidate 2 [Source:ZFIN;Acc:ZDB-GENE-030616-571]
Danio_rerio	10	32015598	32014865	ENSDARG00000004627	PANX3	-1	pannexin 3 [Source:HGNC Symbol;Acc:20573]
Danio_rerio	10	33603205	33606281	ENSDARG00000043285	znf295	1	zinc finger protein 295 [Source:ZFIN;Acc:ZDB-GENE- 050411-7]
Danio_rerio	10	36271933	36270412	ENSDARG00000061378	smg8	-1	smg-8 homolog, nonsense mediated mRNA decay factor (C. elegans) [Source:ZFIN;Acc:ZDB-GENE-091204-279]
Danio_rerio	11	10908119	10908000	ENSDARG00000063100	psmd14	-1	proteasome (prosome, macropain) 26S subunit, non- ATPase, 14 [Source:ZFIN;Acc:ZDB-GENE-070410-56]
Danio_rerio	11	12311921	12312121	ENSDARG00000044943	npepps	1	aminopeptidase puromycin sensitive [Source:ZFIN;Acc:ZDB-GENE-060524-3]
Danio_rerio	11	1354895	1354739	ENSDARG00000007955	iars	-1	isoleucyl-tRNA synthetase [Source:ZFIN;Acc:ZDB-GENE- 030131-6325]

Organisms	Chromosome /Scaffolds	Start Position	Ending Position	Ensembl code	Short name	Strand	Annotation
Danio_rerio	11	16233723	16233931	ENSDARG00000040238	pank4	1	pantothenate kinase 4 [Source:ZFIN;Acc:ZDB-GENE- 040426-1592]
Danio_rerio	11	19924211	19924416	ENSDARG00000070674	psmd6	1	proteasome (prosome, macropain) 26S subunit, non- ATPase, 6 [Source:ZFIN;Acc:ZDB-GENE-040426-1038]
Danio_rerio	11	2584367	2584488	ENSDARG00000091478	DNAJC14	1	DnaJ (Hsp40) homolog, subfamily C, member 14 [Source:HGNC Symbol;Acc:24581]
Danio_rerio	11	28697579	28697189	ENSDARG00000044820	cstf1	-1	cleavage stimulation factor, 3' pre-RNA, subunit 1 [Source:ZFIN;Acc:ZDB-GENE-030131-2958]
Danio_rerio	11	3328880	3327408	ENSDARG00000091574	SUOX	-1	sulfite oxidase [Source:HGNC Symbol;Acc:11460]
Danio_rerio	11	42133756	42134630	ENSDARG00000004302	SLC45A1	1	solute carrier family 45, member 1 [Source:HGNC Symbol;Acc:17939]
Danio_rerio	12	14813631	14813815	ENSDARG00000045123	CR847562.1	1	5-oxoprolinase [Source:RefSeq peptide;Acc:NP_001186988]
Danio_rerio	12	18976269	18976599	ENSDARG00000090442	TRRAP	1	transformation/transcription domain-associated protein [Source:HGNC Symbol;Acc:12347]
Danio_rerio	12	33347692	33347836	ENSDARG00000074121	DNMBP	1	dynamin binding protein [Source:HGNC Symbol;Acc:30373]
Danio_rerio	12	35309185	35308370	ENSDARG00000079969	C12H10orf2	-1	chromosome 10 open reading frame 2 [Source:HGNC Symbol;Acc:1160]
Danio_rerio	12	5636495	5635748	ENSDARG00000063268	SLC35G1	-1	solute carrier family 35, member G1 [Source:HGNC Symbol;Acc:26607]
Danio_rerio	13	12735037	12735832	ENSDARG00000062330	si:ch211-233a24.2	1	si:ch211-233a24.2 [Source:ZFIN;Acc:ZDB-GENE-090313- 98]
Danio_rerio	13	1470585	1472053	ENSDARG00000024748	DST	1	dystonin [Source:HGNC Symbol;Acc:1090]
Danio_rerio	13	18463225	18463413	ENSDARG00000074759	ccar1	1	cell division cycle and apoptosis regulator 1 [Source:ZFIN;Acc:ZDB-GENE-030131-146]
Danio_rerio	13	19367795	19368185	ENSDARG00000039701	emx2	1	empty spiracles homeobox 2 [Source:ZFIN;Acc:ZDB- GENE-990415-54]
Danio_rerio	13	24125183	24125443	ENSDARG00000016855	sf3b5	1	splicing factor 3b, subunit 5 [Source:ZFIN;Acc:ZDB- GENE-040718-181]
Danio_rerio	13	25816686	25816430	ENSDARG00000055305	ret	-1	ret proto-oncogene receptor tyrosine kinase [Source:ZFIN;Acc:ZDB-GENE-980526-307]
Danio_rerio	13	37189920	37189707	ENSDARG00000006196	sav1	-1	salvador homolog 1 (Drosophila) [Source:ZFIN;Acc:ZDB-GENE-040912-28]
Danio_rerio	13	4255675	4255861	ENSDARG00000003449	pde10a	1	phosphodiesterase 10A [Source:ZFIN;Acc:ZDB-GENE- 040426-1115]

Organisms	Chromosome /Scaffolds	Start Position	Ending Position	Ensembl code	Short name	Strand	Annotation
Danio_rerio	13	43870367	43870489	ENSDARG00000007561	CDH23	1	cadherin-related 23 [Source:HGNC Symbol;Acc:13733]
Danio_rerio	13	43891061	43890928	ENSDARG00000006729	eif3s6ip	-1	eukaryotic translation initiation factor 3, subunit 6 interacting protein [Source:ZFIN;Acc:ZDB-GENE-040426- 2138]
Danio_rerio	13	4628267	4628467	ENSDARG00000063358	micu1	1	mitochondrial calcium uptake 1 [Source:ZFIN;Acc:ZDB- GENE-070410-22]
Danio_rerio	13	9224282	9224136	ENSDARG00000028748	tm9sf3	-1	transmembrane 9 superfamily member 3 [Source:ZFIN;Acc:ZDB-GENE-040426-2714]
Danio_rerio	14	19578453	19577740	ENSDARG00000029452	NEURL1B	-1	neuralized homolog 1B (Drosophila) [Source:HGNC Symbol;Acc:35422]
Danio_rerio	14	27116473	27116010	ENSDARG00000018923	FAT2	-1	FAT tumor suppressor homolog 2 (Drosophila) [Source:HGNC Symbol;Acc:3596]
Danio_rerio	14	29479868	29479650	ENSDARG00000061527	nxt2	-1	nuclear transport factor 2-like export factor 2 [Source:ZFIN;Acc:ZDB-GENE-050521-1]
Danio_rerio	14	33713005	33715226	ENSDARG00000055040	nkrf	1	NF-kappa B repressing factor [Source:ZFIN;Acc:ZDB- GENE-030616-73]
Danio_rerio	14	40053968	40054243	ENSDARG00000006924	FBXO38	1	F-box protein 38 [Source:HGNC Symbol;Acc:28844]
Danio_rerio	14	40647984	40648160	ENSDARG00000076060	diaph2	1	diaphanous homolog 2 (Drosophila) [Source:ZFIN;Acc:ZDB-GENE-090115-1]
Danio_rerio	14	47546722	47546937	ENSDARG00000079512	fam193b	1	family with sequence similarity 193, member B [Source:ZFIN;Acc:ZDB-GENE-111102-1]
Danio_rerio	14	53583886	53584025	ENSDARG00000012592	polr2b	1	polymerase (RNA) II (DNA directed) polypeptide B [Source:ZFIN;Acc:ZDB-GENE-041008-1]
Danio_rerio	15	1565400	1565632	ENSDARG00000038882	smc4	1	structural maintenance of chromosomes 4 [Source:ZFIN;Acc:ZDB-GENE-020419-21]
Danio_rerio	15	16110338	16110473	ENSDARG00000018973	clptm1	1	cleft lip and palate associated transmembrane protein 1 [Source:ZFIN;Acc:ZDB-GENE-040801-265]
Danio_rerio	15	1881136	1881263	ENSDARG00000027803	sbds	1	Shwachman-Bodian-Diamond syndrome [Source:ZFIN;Acc:ZDB-GENE-040426-1116]

Organisms	Chromosome /Scaffolds	Start Position	Ending Position	Ensembl code	Short name	Strand	Annotation
Danio_rerio	15	44379711	44379543	ENSDARG00000088574	psmd2	-1	proteasome (prosome, macropain) 26S subunit, non- ATPase, 2 [Source:ZFIN;Acc:ZDB-GENE-040426-1480]
Danio_rerio	15	45488172	45488582	ENSDARG00000027602	LRFN1	1	leucine rich repeat and fibronectin type III domain containing 1 [Source:HGNC Symbol;Acc:29290]
Danio_rerio	15	46583426	46583554	ENSDARG00000043886	paf1	1	paf1, RNA polymerase II associated factor, homolog (S. cerevisiae) [Source:ZFIN;Acc:ZDB-GENE-050506-101]
Danio_rerio	15	6713625	6713942	ENSDARG00000090224	RINL	1	Ras and Rab interactor-like [Source:HGNC Symbol;Acc:24795]
Danio_rerio	16	28635206	28633731	ENSDARG00000079751	megf8	-1	multiple EGF-like-domains 8 [Source:ZFIN;Acc:ZDB- GENE-090730-1]
Danio_rerio	16	29445638	29444739	ENSDARG00000070399	alg2	-1	asparagine-linked glycosylation 2 homolog (S. cerevisiae, alpha-1,3-mannosyltransferase) [Source:ZFIN;Acc:ZDB-GENE-060502-2]
Danio_rerio	16	34146314	34146491	ENSDARG00000011405	rps9	1	ribosomal protein S9 [Source:ZFIN;Acc:ZDB-GENE- 010724-15]
Danio_rerio	16	35393839	35391733	ENSDARG00000019962	dopey1	-1	dopey family member 1 [Source:ZFIN;Acc:ZDB-GENE- 050309-65]
Danio_rerio	16	46147220	46147058	ENSDARG00000017190	strumpellin	-1	strumpellin [Source:ZFIN;Acc:ZDB-GENE-040426-838]
Danio_rerio	16	50507492	50506190	ENSDARG00000010995	mios	-1	missing oocyte, meiosis regulator, homolog (Drosophila) [Source:ZFIN;Acc:ZDB-GENE-040426-856]
Danio_rerio	16	55608807	55608626	ENSDARG00000009480	pdcl	-1	phosducin-like [Source:ZFIN;Acc:ZDB-GENE-030131- 2365]
Danio_rerio	16	56182262	56181994	ENSDARG00000018192	ubr5	-1	ubiquitin protein ligase E3 component n-recognin 5 [Source:ZFIN;Acc:ZDB-GENE-030131-6559]
Danio_rerio	16	56469514	56469382	ENSDARG00000017143	brd9	-1	bromodomain containing 9 [Source:ZFIN;Acc:ZDB-GENE- 060502-1]
Danio_rerio	16	567906	567280	ENSDARG00000001785	irx2a	-1	iroquois homeobox protein 2, a [Source:ZFIN;Acc:ZDB- GENE-040426-1446]
Danio_rerio	16	7946469	7949094	ENSDARG00000087536	ZNF407	1	zinc finger protein 407 [Source:HGNC Symbol;Acc:19904]
Danio_rerio	16	8603325	8603452	ENSDARG00000023396	atg5	1	ATG5 autophagy related 5 homolog (S. cerevisiae) [Source:ZFIN;Acc:ZDB-GENE-040801-149]
Danio_rerio	17	232056	230980	ENSDARG00000014717	DYNC1H1	-1	dynein, cytoplasmic 1, heavy chain 1 [Source:HGNC Symbol;Acc:2961]
Danio_rerio	17	24439918	24440142	ENSDARG00000044090	zmpste24	1	zinc metallopeptidase, STE24 homolog [Source:ZFIN;Acc:ZDB-GENE-030131-6312]

Organisms	Chromosome /Scaffolds	Start Position	Ending Position	Ensembl code	Short name	Strand	Annotation
Danio_rerio	17	25269830	25269952	ENSDARG00000001244	srrm1	1	serine/arginine repetitive matrix 1 [Source:ZFIN;Acc:ZDB-GENE-040426-2789]
Danio_rerio	17	28747512	28748348	ENSDARG00000054213	hectd1	1	HECT domain containing 1 [Source:ZFIN;Acc:ZDB-GENE- 030616-153]
Danio_rerio	17	43127509	43127383	ENSDARG00000053517	EML5	-1	echinoderm microtubule associated protein like 5 [Source:HGNC Symbol;Acc:18197]
Danio_rerio	17	48375123	48373684	ENSDARG00000067841	CABZ01076817.1	-1	Uncharacterized protein [Source:UniProtKB/TrEMBL;Acc:E7FBL7]
Danio_rerio	17	51577517	51577330	ENSDARG00000016775	aqr	-1	aquarius homolog (mouse) [Source:ZFIN;Acc:ZDB-GENE- 040426-1189]
Danio_rerio	17	7445971	7446131	ENSDARG00000087784	BX511121.1	1	Uncharacterized protein [Source:UniProtKB/TrEMBL;Acc:E7F822]
Danio_rerio	18	14979232	14979090	ENSDARG00000062915	polr3b	-1	polymerase (RNA) III (DNA directed) polypeptide B [Source:ZFIN;Acc:ZDB-GENE-030131-2887]
Danio_rerio	18	24758121	24757985	ENSDARG00000060687	chd2	-1	chromodomain helicase DNA binding protein 2 [Source:ZFIN;Acc:ZDB-GENE-050419-256]
Danio_rerio	18	26262685	26262825	ENSDARG00000087485	CABZ01036951.1	1	Uncharacterized protein [Source:UniProtKB/TrEMBL;Acc:E7EZB1]
Danio_rerio	18	36679109	36678938	ENSDARG00000054259	nat10	-1	N-acetyltransferase 10 [Source:ZFIN;Acc:ZDB-GENE- 040426-1543]
Danio_rerio	18	39593040	39593165	ENSDARG00000061397	trip12	1	thyroid hormone receptor interactor 12 [Source:ZFIN;Acc:ZDB-GENE-041111-262]
Danio_rerio	18	7973745	7973508	ENSDARG00000042249	FRMD4A	-1	FERM domain containing 4A [Source:HGNC Symbol;Acc:25491]
Danio_rerio	19	12564665	12564526	ENSDARG00000034178	cpsf1	-1	cleavage and polyadenylation specific factor 1 [Source:ZFIN;Acc:ZDB-GENE-040709-2]
Danio_rerio	19	28452445	28451738	ENSDARG00000079276	UBE2QL1	-1	ubiquitin-conjugating enzyme E2Q family-like 1 [Source:HGNC Symbol;Acc:37269]
Danio_rerio	19	32146188	32142653	ENSDARG00000061804	si:ch211-194e15.5	-1	si:ch211-194e15.5 [Source:ZFIN;Acc:ZDB-GENE-060503- 393]
Danio_rerio	19	32777735	32778436	ENSDARG00000061723	tmem64	1	transmembrane protein 64 [Source:ZFIN;Acc:ZDB-GENE- 060503-182]
Danio_rerio	19	32828385	32828257	ENSDARG00000093406	C19H6orf62	-1	chromosome 6 open reading frame 62 [Source:HGNC Symbol;Acc:20998]

Organisms	Chromosome /Scaffolds	Start Position	Ending Position	Ensembl code	Short name	Strand	Annotation
Danio_rerio	19	41437561	41437701	ENSDARG00000002880	CCDC132=(1=of=2)	1	coiled-coil domain containing 132 [Source:HGNC Symbol;Acc:25956]
Danio_rerio	19	47963705	47963518	ENSDARG00000028249	utp23	-1	UTP23, small subunit (SSU) processome component, homolog (yeast) [Source:ZFIN;Acc:ZDB-GENE-050417-353]
Danio_rerio	19	4938142	4938725	ENSDARG00000094353	TRAPPC9=(2=of=2)	1	trafficking protein particle complex 9 [Source:HGNC Symbol;Acc:30832]
Danio_rerio	19	49803401	49803177	ENSDARG00000008680	kpnb1	-1	karyopherin (importin) beta 1 [Source:ZFIN;Acc:ZDB- GENE-030131-2579]
Danio_rerio	19	7910213	7910049	ENSDARG00000016721	sdha	-1	succinate dehydrogenase complex, subunit A, flavoprotein (Fp) [Source:ZFIN;Acc:ZDB-GENE-040426-874]
Danio_rerio	20	15058321	15055829	ENSDARG00000057583	prrc2c	-1	proline-rich coiled-coil 2C [Source:ZFIN;Acc:ZDB- GENE-081104-156]
Danio_rerio	20	2568697	2568492	ENSDARG00000029157	med23	-1	mediator complex subunit 23 [Source:ZFIN;Acc:ZDB- GENE-040724-82]
Danio_rerio	20	30961284	30961102	ENSDARG00000042642	wtap	-1	Wilms tumor 1 associated protein [Source:ZFIN;Acc:ZDB-GENE-030131-5990]
Danio_rerio	20	32523380	32523545	ENSDARG00000069808	ostm1	1	osteopetrosis associated transmembrane protein 1 [Source:ZFIN;Acc:ZDB-GENE-081104-270]
Danio_rerio	20	33334104	33334754	ENSDARG00000006837	mycn	1	v-myc myelocytomatosis viral related oncogene, neuroblastoma derived (avian) [Source:ZFIN;Acc:ZDB-GENE- 020711-1]
Danio_rerio	20	39322381	39323424	ENSDARG00000008060	scara3	1	scavenger receptor class A, member 3 [Source:ZFIN;Acc:ZDB-GENE-041014-317]
Danio_rerio	20	4037479	4035425	ENSDARG00000063211	exoc8	-1	exocyst complex component 8 [Source:ZFIN;Acc:ZDB- GENE-070410-60]
Danio_rerio	20	50273765	50271624	ENSDARG00000026811	extl3	-1	exostoses (multiple)-like 3 [Source:ZFIN;Acc:ZDB- GENE-041124-2]
Danio_rerio	20	7729733	7729565	ENSDARG00000059925	USP24	-1	ubiquitin specific peptidase 24 [Source:HGNC Symbol;Acc:12623]
Danio_rerio	21	18734269	18731416	ENSDARG00000079104	mfhas1	-1	malignant fibrous histiocytoma amplified sequence 1 [Source:ZFIN;Acc:ZDB-GENE-080917-20]
Danio_rerio	21	21977503	21977310	ENSDARG00000044622	POLQ	-1	polymerase (DNA directed), theta [Source:HGNC Symbol;Acc:9186]

Organisms	Chromosome /Scaffolds	Start Position	Ending Position	Ensembl code	Short name	Strand	Annotation
Danio_rerio	21	23819851	23817617	ENSDARG00000074184	ARHGAP32	-1	Rho GTPase activating protein 32 [Source:HGNC Symbol;Acc:17399]
Danio_rerio	21	25467001	25467177	ENSDARG00000006316	rpl23a	1	ribosomal protein L23a [Source:ZFIN;Acc:ZDB-GENE- 030131-7479]
Danio_rerio	21	25811676	25811172	ENSDARG00000077860	ankhd1	-1	ankyrin repeat and KH domain containing 1 [Source:ZFIN;Acc:ZDB-GENE-090421-1]
Danio_rerio	21	4793806	4792232	ENSDARG00000076991	dolk	-1	dolichol kinase [Source:ZFIN;Acc:ZDB-GENE-070410- 59]
Danio_rerio	21	8560567	8561644	ENSDARG00000060175	ARHGAP24	1	Rho GTPase activating protein 24 [Source:HGNC Symbol;Acc:25361]
Danio_rerio	21	8854506	8854649	ENSDARG00000073862	ptpn13	1	protein tyrosine phosphatase, non-receptor type 13 [Source:ZFIN;Acc:ZDB-GENE-070410-72]
Danio_rerio	21	9124967	9124663	ENSDARG00000057080	dnajc21	-1	DnaJ (Hsp40) homolog, subfamily C, member 21 [Source:ZFIN;Acc:ZDB-GENE-030131-8928]
Danio_rerio	22	10493392	10493264	ENSDARG00000043077	nisch	-1	nischarin [Source:ZFIN;Acc:ZDB-GENE-050208-570]
Danio_rerio	22	10709733	10709595	ENSDARG00000063051	bap1	-1	BRCA1 associated protein-1 (ubiquitin carboxy- terminal hydrolase) [Source:ZFIN;Acc:ZDB-GENE-050208-492]
Danio_rerio	22	11642231	11642790	ENSDARG00000032087	sgsh	1	N-sulfoglucosamine sulfohydrolase (sulfamidase) [Source:ZFIN;Acc:ZDB-GENE-030131-4958]
Danio_rerio	22	19292311	19291520	ENSDARG00000061658	POLRMT	-1	polymerase (RNA) mitochondrial (DNA directed) [Source:HGNC Symbol;Acc:9200]
Danio_rerio	22	32341510	32341682	ENSDARG00000071046	bbip1	1	BBSome interacting protein 1 [Source:ZFIN;Acc:ZDB- GENE-050208-445]
Danio_rerio	22	449972	449838	ENSDARG00000000853	dstyk	-1	dual serine/threonine and tyrosine protein kinase [Source:ZFIN;Acc:ZDB-GENE-040826-2]
Danio_rerio	22	71087	70887	ENSDARG00000090150	CABZ01072698.1	-1	Uncharacterized protein [Source:UniProtKB/TrEMBL;Acc:F1R0D6]
Danio_rerio	23	21647889	21648128	ENSDARG00000009549	ubr4	1	ubiquitin protein ligase E3 component n-recognin 4 [Source:ZFIN;Acc:ZDB-GENE-090313-341]
Danio_rerio	23	31998605	31998456	ENSDARG00000036776	aldh8a1	-1	aldehyde dehydrogenase 8 family, member A1 [Source:ZFIN;Acc:ZDB-GENE-040912-3]
Danio_rerio	23	37174667	37174527	ENSDARG00000025212	cpsf3l	-1	cleavage and polyadenylation specific factor 3-like

Organisms	Chromosome /Scaffolds	Start Position	Ending Position	Ensembl code	Short name	Strand	Annotation
							[Source:ZFIN;Acc:ZDB-GENE-050522-13]
Danio_rerio	23	43795143	43797435	ENSDARG00000004114	tti1	1	TELO2 interacting protein 1 [Source:ZFIN;Acc:ZDB- GENE-080819-3]
Danio_rerio	23	9188003	9188283	ENSDARG00000063089	C23H20orf160	1	chromosome 20 open reading frame 160 [Source:HGNC Symbol;Acc:16153]
Danio_rerio	24	19794000	19794144	ENSDARG00000095034	C24H8orf34	1	chromosome 8 open reading frame 34 [Source:HGNC Symbol;Acc:30905]
Danio_rerio	24	21785506	21785642	ENSDARG00000027825	naa50	1	N(alpha)-acetyltransferase 50, NatE catalytic subunit [Source:ZFIN;Acc:ZDB-GENE-040801-142]
Danio_rerio	24	24325336	24325570	ENSDARG00000057624	cops5	1	COP9 constitutive photomorphogenic homolog subunit 5 [Source:ZFIN;Acc:ZDB-GENE-040426-1686]
Danio_rerio	24	3549666	3549349	ENSDARG00000019976	idi1	-1	isopentenyl-diphosphate delta isomerase 1 [Source:ZFIN;Acc:ZDB-GENE-050913-44]
Danio_rerio	24	37195921	37195797	ENSDARG00000028771	MAPRE2	-1	microtubule-associated protein, RP/EB family, member 2 [Source:HGNC Symbol;Acc:6891]
Danio_rerio	24	8299296	8299142	ENSDARG00000045437	slc35b3	-1	solute carrier family 35, member B3 [Source:ZFIN;Acc:ZDB-GENE-060312-46]
Danio_rerio	25	13645305	13645496	ENSDARG00000006145	det1	1	de-etiolated homolog 1 (Arabidopsis) [Source:ZFIN;Acc:ZDB-GENE-030131-2809]
Danio_rerio	25	17697332	17697628	ENSDARG00000004174	cnot1	1	CCR4-NOT transcription complex, subunit 1 [Source:ZFIN;Acc:ZDB-GENE-040915-1]
Danio_rerio	25	20081942	20081819	ENSDARG00000021370	actr6	-1	ARP6 actin-related protein 6 homolog (yeast) [Source:ZFIN;Acc:ZDB-GENE-030131-3543]
Danio_rerio	25	286066	286194	ENSDARG00000031774	pus7	1	pseudouridylate synthase 7 homolog (S. cerevisiae) [Source:ZFIN;Acc:ZDB-GENE-060620-1]
Danio_rerio	25	4882379	4882518	ENSDARG00000075000	ap4e1	1	adaptor-related protein complex 4, epsilon 1 subunit [Source:ZFIN;Acc:ZDB-GENE-061221-3]
Danio_rerio	25	6020714	6020930	ENSDARG00000089546	TUBGCP6	1	tubulin, gamma complex associated protein 6 [Source:HGNC

Organisms	Chromosome /Scaffolds	Start Position	Ending Position	Ensembl code	Short name	Strand	Annotation
							Symbol;Acc:18127]
Danio_rerio	25	7748046	7747861	ENSDARG00000063147	PTDSS2	-1	phosphatidylserine synthase 2 [Source:HGNC Symbol;Acc:15463]
Danio_rerio	25	9235250	9236997	ENSDARG00000052122	rag1	1	recombination activating gene 1 [Source:ZFIN;Acc:ZDB-GENE-990415-234]
Danio_rerio	Zv9_NA237	14407	14225	ENSDARG00000015911	mcm2	-1	MCM2 minichromosome maintenance deficient 2, mitotin (S. cerevisiae) [Source:ZFIN;Acc:ZDB-GENE-020419-24]
Danio_rerio	Zv9_NA372	184977	184818	ENSDARG00000089100	OFCC1	-1	orofacial cleft 1 candidate 1 [Source:HGNC Symbol;Acc:21017]
Danio_rerio	Zv9_NA403	57460	55995	ENSDARG00000086156	SYNPO	-1	synaptopodin [Source:HGNC Symbol;Acc:30672]
Danio_rerio	Zv9_NA880	20944	21069	ENSDARG00000036570	snrpf	1	small nuclear ribonucleoprotein polypeptide F [Source:ZFIN;Acc:ZDB-GENE-040930-9]
Danio_rerio	Zv9_scaffold3504	59064	58935	ENSDARG00000087895	mib	-1	mind bomb [Source:ZFIN;Acc:ZDB-GENE-030404-2]
Gasterosteus_aculeatus	groupI	7545001	7545288	ENSGACG00000008504	PRPF8	1	PRP8 pre-mRNA processing factor 8 homolog (S. cerevisiae) [Source:HGNC Symbol;Acc:17340]
Gasterosteus_aculeatus	groupI	9297590	9297741	ENSGACG00000009507	AP2S1	1	adaptor-related protein complex 2, sigma 1 subunit [Source:HGNC Symbol;Acc:565]
Gasterosteus_aculeatus	groupII	10023301	10022036	ENSGACG00000015658	ST5	-1	suppression of tumorigenicity 5 [Source:HGNC Symbol;Acc:11350]
Gasterosteus_aculeatus	groupII	10483463	10482668	ENSGACG00000015728	USP47	-1	ubiquitin specific peptidase 47 [Source:HGNC Symbol;Acc:20076]
Gasterosteus_aculeatus	groupII	11497785	11498042	ENSGACG00000015833	MNS1	1	meiosis-specific nuclear structural 1 [Source:HGNC Symbol;Acc:29636]
Gasterosteus_aculeatus	groupII	11694540	11695784	ENSGACG00000015882	FTSJD1	1	FtsJ methyltransferase domain containing 1 [Source:HGNC Symbol;Acc:25635]
Gasterosteus_aculeatus	groupII	19418278	19418449	ENSGACG00000017021	BBS4	1	Bardet-Biedl syndrome 4 [Source:HGNC Symbol;Acc:969]
Gasterosteus_aculeatus	groupII	5732862	5732997	ENSGACG00000014999	LEO1	1	Leo1, Paf1/RNA polymerase II complex component, homolog (S. cerevisiae) [Source:HGNC Symbol;Acc:30401]
Gasterosteus_aculeatus	groupIII	16377385	16377147	ENSGACG00000017927	CEBPD	-1	CCAAT/enhancer binding protein (C/EBP), delta [Source:HGNC Symbol;Acc:1835]
Gasterosteus_aculeatus	groupIII	16528366	16526477	ENSGACG00000017953	RB1CC1	-1	RB1-inducible coiled-coil 1 [Source:HGNC Symbol;Acc:15574]
Gasterosteus_aculeatus	groupIII	16627074	16626624	ENSGACG00000017959	GBX1	-1	gastrulation brain homeobox 1 [Source:HGNC Symbol;Acc:4185]

Organisms	Chromosome /Scaffolds	Start Position	Ending Position	Ensembl code	Short name	Strand	Annotation
Gasterosteus_aculeatus	groupIII	7534838	7535419	ENSGACG00000015324	CACTIN	1	cactin, spliceosome C complex subunit [Source:HGNC Symbol;Acc:29938]
Gasterosteus_aculeatus	groupIV	10591307	10591145	ENSGACG00000017960	INTS10	-1	integrator complex subunit 10 [Source:HGNC Symbol;Acc:25548]
Gasterosteus_aculeatus	groupIV	14516687	14515926	ENSGACG00000018484	MED7	-1	mediator complex subunit 7 [Source:HGNC Symbol;Acc:2378]
Gasterosteus_aculeatus	groupIV	20834133	20835191	ENSGACG00000019026	ALG10B	1	asparagine-linked glycosylation 10, alpha-1,2- glucosyltransferase homolog B (yeast) [Source:HGNC Symbol;Acc:31088]
Gasterosteus_aculeatus	groupIV	22552368	22552537	ENSGACG00000019212	TRAPPC11	1	trafficking protein particle complex 11 [Source:HGNC Symbol;Acc:25751]
Gasterosteus_aculeatus	groupIV	25859038	25858788	ENSGACG00000019522	ARID2	-1	AT rich interactive domain 2 (ARID, RFX-like) [Source:HGNC Symbol;Acc:18037]
Gasterosteus_aculeatus	groupIV	29005005	29005177	ENSGACG00000019702	SMO	1	smoothened, frizzled family receptor [Source:HGNC Symbol;Acc:11119]
Gasterosteus_aculeatus	groupIV	29480199	29480436	ENSGACG00000019721	EXOC4	1	exocyst complex component 4 [Source:HGNC Symbol;Acc:30389]
Gasterosteus_aculeatus	groupIV	3817767	3817926	ENSGACG00000016749	SRP72	1	signal recognition particle 72kDa [Source:HGNC Symbol;Acc:11303]
Gasterosteus_aculeatus	groupIV	8520482	8520795	ENSGACG00000017689	TSC22D3	1	TSC22 domain family, member 3 [Source:HGNC Symbol;Acc:3051]
Gasterosteus_aculeatus	groupIX	10318026	10317851	ENSGACG00000018193	RPTOR	-1	regulatory associated protein of MTOR, complex 1 [Source:HGNC Symbol;Acc:30287]
Gasterosteus_aculeatus	groupIX	10730774	10730459	ENSGACG00000018238	TAF5	-1	TAF5 RNA polymerase II, TATA box binding protein (TBP)-associated factor, 100kDa [Source:HGNC Symbol;Acc:11539]
Gasterosteus_aculeatus	groupIX	13986085	13985872	ENSGACG00000018708	SMC3	-1	structural maintenance of chromosomes 3 [Source:HGNC Symbol;Acc:2468]
Gasterosteus_aculeatus	groupIX	15170450	15170817	ENSGACG00000019010	SGSH	1	N-sulfoglucosamine sulfohydrolase [Source:HGNC Symbol;Acc:10818]
Gasterosteus_aculeatus	groupIX	19232781	19232452	ENSGACG00000019735	USP34	-1	ubiquitin specific peptidase 34 [Source:HGNC Symbol;Acc:20066]
Gasterosteus_aculeatus	groupIX	5894096	5896405	ENSGACG00000016951	SART1	1	squamous cell carcinoma antigen recognized by T cells [Source:HGNC Symbol;Acc:10538]
Gasterosteus_aculeatus	groupIX	8651590	8651827	ENSGACG00000017771	GUCY1B3	1	guanylate cyclase 1, soluble, beta 3 [Source:HGNC Symbol;Acc:4687]
Gasterosteus_aculeatus	groupIX	9626580	9625750	ENSGACG00000018024	UGT8	-1	UDP glycosyltransferase 8 [Source:HGNC Symbol;Acc:12555]
Gasterosteus_aculeatus	groupV	10672175	10671914	ENSGACG00000008595	ITFG3	-1	integrin alpha FG-GAP repeat containing 3 [Source:HGNC

Organisms	Chromosome /Scaffolds	Start Position	Ending Position	Ensembl code	Short name	Strand	Annotation
							Symbol;Acc:14163]
Gasterosteus_aculeatus	groupV	121793	122059	ENSGACG00000002103	ZDHHC6	1	zinc finger, DHHC-type containing 6 [Source:HGNC Symbol;Acc:19160]
Gasterosteus_aculeatus	groupV	3707006	3707708	ENSGACG00000003504	C10orf2	1	chromosome 10 open reading frame 2 [Source:HGNC Symbol;Acc:1160]
Gasterosteus_aculeatus	groupV	4644730	4644592	ENSGACG00000003963	SNF8	-1	SNF8, ESCRT-II complex subunit, homolog (S. cerevisiae) [Source:HGNC Symbol;Acc:17028]
Gasterosteus_aculeatus	groupV	4815667	4815856	ENSGACG00000004173	TUBGCP2	1	tubulin, gamma complex associated protein 2 [Source:HGNC Symbol;Acc:18599]
Gasterosteus_aculeatus	groupV	6001202	6001359	ENSGACG00000004961	GET4	1	golgi to ER traffic protein 4 homolog (S. cerevisiae) [Source:HGNC Symbol;Acc:21690]
Gasterosteus_aculeatus	groupV	6334923	6335076	ENSGACG00000005292	C17orf101	1	chromosome 17 open reading frame 101 [Source:HGNC Symbol;Acc:26174]
Gasterosteus_aculeatus	groupV	8066752	8066888	ENSGACG00000006114	GPAM	1	glycerol-3-phosphate acyltransferase, mitochondrial [Source:HGNC Symbol;Acc:24865]
Gasterosteus_aculeatus	groupVI	2224341	2224581	ENSGACG00000002936	BTAF1	1	BTAF1 RNA polymerase II, B-TFIID transcription factor-associated, 170kDa (Mot1 homolog, S. cerevisiae) [Source:HGNC Symbol;Acc:17307]
Gasterosteus_aculeatus	groupVI	6368659	6364893	ENSGACG00000005370	GOLGA4	-1	golgin A4 [Source:HGNC Symbol;Acc:4427]
Gasterosteus_aculeatus	groupVI	7336430	7335677	ENSGACG00000006014	BAI3	-1	brain-specific angiogenesis inhibitor 3 [Source:HGNC Symbol;Acc:945]
Gasterosteus_aculeatus	groupVII	13512188	13511784	ENSGACG00000020203	C12orf44	-1	chromosome 12 open reading frame 44 [Source:HGNC Symbol;Acc:25679]
Gasterosteus_aculeatus	groupVII	14359386	14359569	ENSGACG00000020225	ZZEF1	1	zinc finger, ZZ-type with EF-hand domain 1 [Source:HGNC Symbol;Acc:29027]
Gasterosteus_aculeatus	groupVII	14970557	14971172	ENSGACG00000020252	FAM222B=(1=of=2)	1	family with sequence similarity 222, member B [Source:HGNC Symbol;Acc:25563]
Gasterosteus_aculeatus	groupVII	17570618	17571003	ENSGACG00000020367	THOC2	1	THO complex 2 [Source:HGNC Symbol;Acc:19073]
Gasterosteus_aculeatus	groupVII	22069731	22069000	ENSGACG00000020662	NFRKB	-1	nuclear factor related to kappaB binding protein [Source:HGNC Symbol;Acc:7802]

Organisms	Chromosome /Scaffolds	Start Position	Ending Position	Ensembl code	Short name	Strand	Annotation
Gasterosteus_aculeatus	groupVII	26777478	26776980	ENSGACG00000020846	LIG3	-1	ligase III, DNA, ATP-dependent [Source:HGNC Symbol;Acc:6600]
Gasterosteus_aculeatus	groupVII	26900758	26900894	ENSGACG00000020853	C11orf30	1	chromosome 11 open reading frame 30 [Source:HGNC Symbol;Acc:18071]
Gasterosteus_aculeatus	groupVII	3940452	3941056	ENSGACG00000019304	KIAA1239=(1=of=2)	1	KIAA1239 [Source:HGNC Symbol;Acc:29229]
Gasterosteus_aculeatus	groupVII	4299616	4299823	ENSGACG00000019366	POLR2A	1	polymerase (RNA) II (DNA directed) polypeptide A, 220kDa [Source:HGNC Symbol;Acc:9187]
Gasterosteus_aculeatus	groupVII	4554466	4554147	ENSGACG00000019426	MSANTD1	-1	Myb/SANT-like DNA-binding domain containing 1 [Source:HGNC Symbol;Acc:33741]
Gasterosteus_aculeatus	groupVII	6666937	6667134	ENSGACG00000019601	OSTC	1	oligosaccharyltransferase complex subunit [Source:HGNC Symbol;Acc:24448]
Gasterosteus_aculeatus	groupVIII	12473210	12473363	ENSGACG00000009686	RPF1	1	ribosome production factor 1 homolog (S. cerevisiae) [Source:HGNC Symbol;Acc:30350]
Gasterosteus_aculeatus	groupVIII	14212893	14213111	ENSGACG00000011164	BEND5	1	BEN domain containing 5 [Source:HGNC Symbol;Acc:25668]
Gasterosteus_aculeatus	groupVIII	5635070	5634881	ENSGACG00000005873	GLRX2	-1	glutaredoxin 2 [Source:HGNC Symbol;Acc:16065]
Gasterosteus_aculeatus	groupVIII	7901566	7901734	ENSGACG00000007086	CACHD1	1	cache domain containing 1 [Source:HGNC Symbol;Acc:29314]
Gasterosteus_aculeatus	groupX	12897626	12897797	ENSGACG00000008476	TXNL4A	1	thioredoxin-like 4A [Source:HGNC Symbol;Acc:30551]
Gasterosteus_aculeatus	groupX	1864277	1870053	ENSGACG00000002352	HIVEP1	1	human immunodeficiency virus type I enhancer binding protein 1 [Source:HGNC Symbol;Acc:4920]
Gasterosteus_aculeatus	groupX	9481770	9482013	ENSGACG00000006780	DLX6	1	distal-less homeobox 6 [Source:HGNC Symbol;Acc:2919]
Gasterosteus_aculeatus	groupXI	13673193	13673358	ENSGACG00000013930	NPRL3	1	nitrogen permease regulator-like 3 (S. cerevisiae) [Source:HGNC Symbol;Acc:14124]
Gasterosteus_aculeatus	groupXI	14272044	14271870	ENSGACG00000014088	INTS1	-1	integrator complex subunit 1 [Source:HGNC Symbol;Acc:24555]
Gasterosteus_aculeatus	groupXI	14969672	14969947	ENSGACG00000014382	DEXI	1	Dexi homolog (mouse) [Source:HGNC Symbol;Acc:13267]
Gasterosteus_aculeatus	groupXI	316700	316851	ENSGACG00000004710	ABAT	1	4-aminobutyrate aminotransferase [Source:HGNC Symbol;Acc:23]
Gasterosteus_aculeatus	groupXII	10446925	10446753	ENSGACG00000008262	FANCE	-1	Fanconi anemia, complementation group E [Source:HGNC Symbol;Acc:3586]
Gasterosteus_aculeatus	groupXII	10895298	10895837	ENSGACG00000008789	HUWE1	1	HECT, UBA and WWE domain containing 1, E3 ubiquitin protein ligase

Organisms	Chromosome /Scaffolds	Start Position	Ending Position	Ensembl code	Short name	Strand	Annotation
							[Source:HGNC Symbol;Acc:30892]
Gasterosteus_aculeatus	groupXII	13732754	13732400	ENSGACG00000010838	RNF41	-1	ring finger protein 41 [Source:HGNC Symbol;Acc:18401]
Gasterosteus_aculeatus	groupXII	17646948	17646793	ENSGACG00000013073	RPL10L	-1	ribosomal protein L10-like [Source:HGNC Symbol;Acc:17976]
Gasterosteus_aculeatus	groupXII	8808010	8807761	ENSGACG00000006920	PIGT	-1	phosphatidylinositol glycan anchor biosynthesis, class T [Source:HGNC Symbol;Acc:14938]
Gasterosteus_aculeatus	groupXII	9450172	9449981	ENSGACG00000007229	UBE4B	-1	ubiquitination factor E4B [Source:HGNC Symbol;Acc:12500]
Gasterosteus_aculeatus	groupXIII	12841927	12841799	ENSGACG00000011293	HECTD4=(2=of=2)	-1	HECT domain containing E3 ubiquitin protein ligase 4 [Source:HGNC Symbol;Acc:26611]
Gasterosteus_aculeatus	groupXIII	12961854	12961705	ENSGACG00000011474	SNRNP200	-1	small nuclear ribonucleoprotein 200kDa (U5) [Source:HGNC Symbol;Acc:30859]
Gasterosteus_aculeatus	groupXIII	14421087	14421901	ENSGACG00000012373	CCDC92	1	coiled-coil domain containing 92 [Source:HGNC Symbol;Acc:29563]
Gasterosteus_aculeatus	groupXIII	14515679	14515477	ENSGACG00000012469	CABIN1	-1	calcineurin binding protein 1 [Source:HGNC Symbol;Acc:24187]
Gasterosteus_aculeatus	groupXIII	14813118	14813619	ENSGACG00000012764	CTU1	1	cytosolic thiouridylase subunit 1 homolog (S. pombe) [Source:HGNC Symbol;Acc:29590]
Gasterosteus_aculeatus	groupXIII	15011259	15011019	ENSGACG00000012865	GALNT9	-1	UDP-N-acetyl-alpha-D-galactosamine:polypeptide N-acetylgalactosaminyltransferase 9 (GalNAc-T9) [Source:HGNC Symbol;Acc:4131]
Gasterosteus_aculeatus	groupXIII	18345958	18346114	ENSGACG00000014252	SUDS3	1	suppressor of defective silencing 3 homolog (S. cerevisiae) [Source:HGNC Symbol;Acc:29545]
Gasterosteus_aculeatus	groupXIII	8888462	8889543	ENSGACG00000008784	FICD	1	FIC domain containing [Source:HGNC Symbol;Acc:18416]
Gasterosteus_aculeatus	groupXIII	9321849	9321989	ENSGACG00000009515	MORC2	1	MORC family CW-type zinc finger 2 [Source:HGNC Symbol;Acc:23573]
Gasterosteus_aculeatus	groupXIV	14997880	14997606	ENSGACG00000018644	CHD1	-1	chromodomain helicase DNA binding protein 1 [Source:HGNC Symbol;Acc:1915]
Gasterosteus_aculeatus	groupXIV	2904583	2904754	ENSGACG00000016022	RAPGEF1	1	Rap guanine nucleotide exchange factor (GEF) 1 [Source:HGNC Symbol;Acc:4568]
Gasterosteus_aculeatus	groupXIV	3924728	3926560	ENSGACG00000016456	ZNF532	1	zinc finger protein 532 [Source:HGNC Symbol;Acc:30940]

Organisms	Chromosome /Scaffolds	Start Position	Ending Position	Ensembl code	Short name	Strand	Annotation
Gasterosteus_aculeatus	groupXIV	7366748	7366585	ENSGACG00000017341	NDUFA8	-1	NADH dehydrogenase (ubiquinone) 1 alpha subcomplex, 8, 19kDa [Source:HGNC Symbol;Acc:7692]
Gasterosteus_aculeatus	groupXIX	14489909	14491510	ENSGACG00000011461	RAG2	1	recombination activating gene 2 [Source:HGNC Symbol;Acc:9832]
Gasterosteus_aculeatus	groupXIX	16760830	16760707	ENSGACG00000013064	PTPRQ	-1	protein tyrosine phosphatase, receptor type, Q [Source:HGNC Symbol;Acc:9679]
Gasterosteus_aculeatus	groupXIX	17830007	17828893	ENSGACG00000013460	PANX2	-1	pannexin 2 [Source:HGNC Symbol;Acc:8600]
Gasterosteus_aculeatus	groupXIX	4472606	4471968	ENSGACG00000003562	C7orf60	-1	chromosome 7 open reading frame 60 [Source:HGNC Symbol;Acc:26475]
Gasterosteus_aculeatus	groupXIX	6856985	6857107	ENSGACG00000005265	RAPSN	1	receptor-associated protein of the synapse [Source:HGNC Symbol;Acc:9863]
Gasterosteus_aculeatus	groupXIX	8647102	8643575	ENSGACG00000007480	CSPG4	-1	chondroitin sulfate proteoglycan 4 [Source:HGNC Symbol;Acc:2466]
Gasterosteus_aculeatus	groupXV	14111585	14111745	ENSGACG00000012898	SLC4A1AP	1	solute carrier family 4 (anion exchanger), member 1, adaptor protein [Source:HGNC Symbol;Acc:13813]
Gasterosteus_aculeatus	groupXV	1435452	1435081	ENSGACG00000005118	DCAF10	-1	DDB1 and CUL4 associated factor 10 [Source:HGNC Symbol;Acc:23686]
Gasterosteus_aculeatus	groupXV	1659212	1659072	ENSGACG00000005379	VPS39	-1	vacuolar protein sorting 39 homolog (S. cerevisiae) [Source:HGNC Symbol;Acc:20593]
Gasterosteus_aculeatus	groupXV	1997572	1997424	ENSGACG00000005774	FCF1	-1	FCF1 small subunit (SSU) processome component homolog (S. cerevisiae) [Source:HGNC Symbol;Acc:20220]
Gasterosteus_aculeatus	groupXV	9847056	9847291	ENSGACG00000010787	ATR	1	ataxia telangiectasia and Rad3 related [Source:HGNC Symbol;Acc:882]
Gasterosteus_aculeatus	groupXVI	10906239	10905548	ENSGACG00000005648	TBR1	-1	T-box, brain, 1 [Source:HGNC Symbol;Acc:11590]
Gasterosteus_aculeatus	groupXVI	7719386	7718937	ENSGACG00000003400	PIGA	-1	phosphatidylinositol glycan anchor biosynthesis, class A [Source:HGNC Symbol;Acc:8957]
Gasterosteus_aculeatus	groupXVI	8600325	8600459	ENSGACG00000003798	RPE	1	ribulose-5-phosphate-3-epimerase [Source:HGNC Symbol;Acc:10293]
Gasterosteus_aculeatus	groupXVI	9555772	9554748	ENSGACG00000004314	INO80D	-1	INO80 complex subunit D [Source:HGNC Symbol;Acc:25997]
Gasterosteus_aculeatus	groupXVI	9680696	9680571	ENSGACG00000004444	AGPS	-1	alkylglycerone phosphate synthase [Source:HGNC Symbol;Acc:327]
Gasterosteus_aculeatus	groupXVII	1718678	1721385	ENSGACG00000004202	RBM12	1	RNA binding motif protein 12 [Source:HGNC Symbol;Acc:9898]

Organisms	Chromosome /Scaffolds	Start Position	Ending Position	Ensembl code	Short name	Strand	Annotation
Gasterosteus_aculeatus	groupXVIII	10387949	10387773	ENSGACG00000010546	YLPM1	-1	YLP motif containing 1 [Source:HGNC Symbol;Acc:17798]
Gasterosteus_aculeatus	groupXVIII	11821594	11821738	ENSGACG00000011539	CDC40	1	cell division cycle 40 homolog (S. cerevisiae) [Source:HGNC Symbol;Acc:17350]
Gasterosteus_aculeatus	groupXVIII	12492229	12492059	ENSGACG00000011852	CRNKL1	-1	crooked neck pre-mRNA splicing factor-like 1 (Drosophila) [Source:HGNC Symbol;Acc:15762]
Gasterosteus_aculeatus	groupXVIII	15233288	15233106	ENSGACG00000013159	C1orf95	-1	chromosome 1 open reading frame 95 [Source:HGNC Symbol;Acc:30491]
Gasterosteus_aculeatus	groupXVIII	978292	978023	ENSGACG00000004386	DISP1	-1	dispatched homolog 1 (Drosophila) [Source:HGNC Symbol;Acc:19711]
Gasterosteus_aculeatus	groupXX	10598097	10598927	ENSGACG00000009027	NR4A3	1	nuclear receptor subfamily 4, group A, member 3 [Source:HGNC Symbol;Acc:7982]
Gasterosteus_aculeatus	groupXX	13427022	13426822	ENSGACG00000012292	ADAM22	-1	ADAM metallopeptidase domain 22 [Source:HGNC Symbol;Acc:201]
Gasterosteus_aculeatus	groupXX	158613	159330	ENSGACG00000002977	PIK3R4	1	phosphoinositide-3-kinase, regulatory subunit 4 [Source:HGNC Symbol;Acc:8982]
Gasterosteus_aculeatus	groupXX	2430566	2432375	ENSGACG00000004511	NOD1	1	nucleotide-binding oligomerization domain containing 1 [Source:HGNC Symbol;Acc:16390]
Gasterosteus_aculeatus	groupXX	3230241	3229727	ENSGACG00000004972	FBXL4	-1	F-box and leucine-rich repeat protein 4 [Source:HGNC Symbol;Acc:13601]
Gasterosteus_aculeatus	groupXX	3323166	3324634	ENSGACG00000005032	PRDM13	1	PR domain containing 13 [Source:HGNC Symbol;Acc:13998]
Gasterosteus_aculeatus	groupXX	4432860	4433158	ENSGACG00000005544	MBP	1	myelin basic protein [Source:HGNC Symbol;Acc:6925]
Gasterosteus_aculeatus	groupXXI	7005783	7005410	ENSGACG00000002837	SULF1	-1	sulfatase 1 [Source:HGNC Symbol;Acc:20391]
Gasterosteus_aculeatus	groupXXI	7725104	7723318	ENSGACG00000003204	KIAA2018	-1	KIAA2018 [Source:HGNC Symbol;Acc:30494]
Gasterosteus_aculeatus	groupXXI	8714495	8714364	ENSGACG00000003838	KIAA1217	-1	KIAA1217 [Source:HGNC Symbol;Acc:25428]
Gasterosteus_aculeatus	scaffold_106	333563	333072	ENSGACG00000001686	PAQR9	-1	progestin and adipoQ receptor family member IX [Source:HGNC Symbol;Acc:30131]
Gasterosteus_aculeatus	scaffold_121	150289	149510	ENSGACG00000001080	UPF2	-1	UPF2 regulator of nonsense transcripts homolog (yeast) [Source:HGNC Symbol;Acc:17854]
Gasterosteus_aculeatus	scaffold_156	34534	34319	ENSGACG00000001281	ANAPC1	-1	anaphase promoting complex subunit 1 [Source:HGNC

Organisms	Chromosome /Scaffolds	Start Position	Ending Position	Ensembl code	Short name	Strand	Annotation
							Symbol;Acc:19988]
Gasterosteus_aculeatus	scaffold_180	19760	18492	ENSGACG00000001600	NCOA6	-1	nuclear receptor coactivator 6 [Source:HGNC Symbol;Acc:15936]
Gasterosteus_aculeatus	scaffold_211	77905	77750	ENSGACG00000011116	DHCR24	-1	24-dehydrocholesterol reductase [Source:HGNC Symbol;Acc:2859]
Gasterosteus_aculeatus	scaffold_27	2771204	2771632	ENSGACG00000000930	THUMPD3	1	THUMP domain containing 3 [Source:HGNC Symbol;Acc:24493]
Gasterosteus_aculeatus	scaffold_324	12639	12418	ENSGACG00000000166	DDX46	-1	DEAD (Asp-Glu-Ala-Asp) box polypeptide 46 [Source:HGNC Symbol;Acc:18681]
Gasterosteus_aculeatus	scaffold_37	2069910	2068724	ENSGACG00000000999	ZNF438	-1	zinc finger protein 438 [Source:HGNC Symbol;Acc:21029]
Gasterosteus_aculeatus	scaffold_47	1540967	1539756	ENSGACG00000000960	TADA2B	-1	transcriptional adaptor 2B [Source:HGNC Symbol;Acc:30781]
Gasterosteus_aculeatus	scaffold_90	160746	159472	ENSGACG00000000611	GPR19	-1	G protein-coupled receptor 19 [Source:HGNC Symbol;Acc:4473]
Gasterosteus_aculeatus	scaffold_98	40443	41507	ENSGACG00000012450	MESDC1	1	mesoderm development candidate 1 [Source:HGNC Symbol;Acc:13519]
Oryzias_latipes	1	14410545	14410422	ENSORLG00000004042	RNASEH2A	-1	ribonuclease H2, subunit A [Source:HGNC Symbol;Acc:18518]
Oryzias_latipes	1	19523985	19522215	ENSORLG00000006383	MOGS	-1	mannosyl-oligosaccharide glucosidase [Source:HGNC Symbol;Acc:24862]
Oryzias_latipes	1	21481398	21481529	ENSORLG00000007283	HERC3	1	HECT and RLD domain containing E3 ubiquitin protein ligase 3 [Source:HGNC Symbol;Acc:4876]
Oryzias_latipes	1	2473136	2472909	ENSORLG00000000849	SRP68	-1	signal recognition particle 68kDa [Source:HGNC Symbol;Acc:11302]
Oryzias_latipes	1	24751546	24754136	ENSORLG00000008328	DCHS2	1	dachsous 2 (Drosophila) [Source:HGNC Symbol;Acc:23111]
Oryzias_latipes	2	5013211	5013059	ENSORLG00000000063	oleed	-1	embryonic ectoderm development protein [Source:RefSeq peptide;Acc:NP_001098326]
Oryzias_latipes	3	14100343	14100202	ENSORLG00000004502	TDRD3	-1	tudor domain containing 3 [Source:HGNC Symbol;Acc:20612]
Oryzias_latipes	3	16126437	16126196	ENSORLG00000005689	SPG7	-1	spastic paraplegia 7 (pure and complicated autosomal recessive) [Source:HGNC Symbol;Acc:11237]
Oryzias_latipes	3	19258428	19258194	ENSORLG00000007874	ZFHX3	-1	zinc finger homeobox 3 [Source:HGNC Symbol;Acc:777]
Oryzias_latipes	3	19970055	19970421	ENSORLG00000008161	SLTM	1	SAFB-like, transcription modulator [Source:HGNC Symbol;Acc:20709]
Oryzias_latipes	3	29568051	29568186	ENSORLG00000013923	BRD7	1	bromodomain containing 7 [Source:HGNC Symbol;Acc:14310]
Oryzias_latipes	3	32577926	32577701	ENSORLG00000015091	NETO2=(1=of=2)	-1	neuropilin (NRP) and tolloid (TLL)-like 2 [Source:HGNC Symbol;Acc:14644]

Organisms	Chromosome /Scaffolds	Start Position	Ending Position	Ensembl code	Short name	Strand	Annotation
Oryzias_latipes	3	8345246	8345041	ENSORLG00000001286	NR1H3	-1	nuclear receptor subfamily 1, group H, member 3 [Source:HGNC Symbol;Acc:7966]
Oryzias_latipes	4	30485115	30485330	ENSORLG00000016163	FBXL5	1	F-box and leucine-rich repeat protein 5 [Source:HGNC Symbol;Acc:13602]
Oryzias_latipes	4	30974028	30975464	ENSORLG00000016350	RAI2	1	retinoic acid induced 2 [Source:HGNC Symbol;Acc:9835]
Oryzias_latipes	4	4420702	4422307	ENSORLG00000001937	KIAA1211	1	KIAA1211 [Source:HGNC Symbol;Acc:29219]
Oryzias_latipes	5	11628455	11628186	ENSORLG00000006234	PXMP4	-1	peroxisomal membrane protein 4, 24kDa [Source:HGNC Symbol;Acc:15920]
Oryzias_latipes	5	15202613	15201794	ENSORLG00000008029	KBTBD8	-1	kelch repeat and BTB (POZ) domain containing 8 [Source:HGNC Symbol;Acc:30691]
Oryzias_latipes	5	19078540	19078361	ENSORLG00000010336	USP48	-1	ubiquitin specific peptidase 48 [Source:HGNC Symbol;Acc:18533]
Oryzias_latipes	5	27584918	27584784	ENSORLG00000014746	TH1L	-1	TH1-like (Drosophila) [Source:HGNC Symbol;Acc:15934]
Oryzias_latipes	5	33773489	33773346	ENSORLG00000017459	PIGU	-1	phosphatidylinositol glycan anchor biosynthesis, class U [Source:HGNC Symbol;Acc:15791]
Oryzias_latipes	5	9459649	9459772	ENSORLG00000005502	R3HDM2	1	R3H domain containing 2 [Source:HGNC Symbol;Acc:29167]
Oryzias_latipes	6	19750191	19750335	ENSORLG00000012869	MBTPS1	1	membrane-bound transcription factor peptidase, site 1 [Source:HGNC Symbol;Acc:15456]
Oryzias_latipes	7	11945142	11945428	ENSORLG00000007137	WNK3	1	WNK lysine deficient protein kinase 3 [Source:HGNC Symbol;Acc:14543]
Oryzias_latipes	7	17385038	17386225	ENSORLG00000010969	PLXND1	1	plexin D1 [Source:HGNC Symbol;Acc:9107]
Oryzias_latipes	7	636033	635833	ENSORLG00000000484	MTOR	-1	mechanistic target of rapamycin (serine/threonine kinase) [Source:HGNC Symbol;Acc:3942]
Oryzias_latipes	7	6457788	6458666	ENSORLG00000002833	XPC	1	xeroderma pigmentosum, complementation group C [Source:HGNC Symbol;Acc:12816]
Oryzias_latipes	7	9637619	9638063	ENSORLG00000005154	PEX14	1	peroxisomal biogenesis factor 14 [Source:HGNC Symbol;Acc:8856]
Oryzias_latipes	8	11148584	11148325	ENSORLG00000008142	ABCA3	-1	ATP-binding cassette, sub-family A (ABC1), member 3 [Source:HGNC Symbol;Acc:33]

Organisms	Chromosome /Scaffolds	Start Position	Ending Position	Ensembl code	Short name	Strand	Annotation
Oryzias_latipes	8	15094086	15094259	ENSORLG00000011718	MAPK8IP3	1	mitogen-activated protein kinase 8 interacting protein 3 [Source:HGNC Symbol;Acc:6884]
Oryzias_latipes	8	6775553	6775350	ENSORLG00000004426	TUBG2	-1	tubulin, gamma 2 [Source:HGNC Symbol;Acc:12419]
Oryzias_latipes	9	11144262	11144683	ENSORLG00000005629	BRI3BP	1	BRI3 binding protein [Source:HGNC Symbol;Acc:14251]
Oryzias_latipes	9	12731860	12732108	ENSORLG00000007310	GIT2=(1=of=2)	1	G protein-coupled receptor kinase interacting ArfGAP 2 [Source:HGNC Symbol;Acc:4273]
Oryzias_latipes	9	12956383	12956685	ENSORLG00000007834	POLE	1	polymerase (DNA directed), epsilon, catalytic subunit [Source:HGNC Symbol;Acc:9177]
Oryzias_latipes	9	2010324	2010471	ENSORLG00000000914	FBXO21	1	F-box protein 21 [Source:HGNC Symbol;Acc:13592]
Oryzias_latipes	9	20150042	20151355	ENSORLG00000013902	EP400	1	E1A binding protein p400 [Source:HGNC Symbol;Acc:11958]
Oryzias_latipes	9	23337195	23337830	ENSORLG00000015210	ARVCF	1	armadillo repeat gene deleted in velocardiofacial syndrome [Source:HGNC Symbol;Acc:728]
Oryzias_latipes	9	2899473	2900962	ENSORLG00000001210	ZNRF3	1	zinc and ring finger 3 [Source:HGNC Symbol;Acc:18126]
Oryzias_latipes	9	510018	510221	ENSORLG00000000299	KREMEN2	1	kringle containing transmembrane protein 2 [Source:HGNC Symbol;Acc:18797]
Oryzias_latipes	9	9039709	9039257	ENSORLG00000004879	GRID2	-1	glutamate receptor, ionotropic, delta 2 [Source:HGNC Symbol;Acc:4576]
Oryzias_latipes	10	14233039	14235746	ENSORLG00000006198	FRMPD3	1	FERM and PDZ domain containing 3 [Source:HGNC Symbol;Acc:29382]
Oryzias_latipes	10	6811316	6812001	ENSORLG00000001659	GPR101	1	G protein-coupled receptor 101 [Source:HGNC Symbol;Acc:14963]
Oryzias_latipes	10	6836748	6837064	ENSORLG00000001692	RBMXL3	1	RNA binding motif protein, X-linked-like 3 [Source:HGNC Symbol;Acc:26859]
Oryzias_latipes	11	17363490	17363654	ENSORLG00000008175	SF3A3	1	splicing factor 3a, subunit 3, 60kDa [Source:HGNC Symbol;Acc:10767]
Oryzias_latipes	11	9811901	9811544	ENSORLG00000004561	DLX5	-1	distal-less homeobox 5 [Source:HGNC Symbol;Acc:2918]
Oryzias_latipes	12	2022363	2022540	ENSORLG00000001971	ADAMTS6	1	ADAM metallopeptidase with thrombospondin type 1 motif, 6 [Source:HGNC Symbol;Acc:222]
Oryzias_latipes	12	24353700	24353876	ENSORLG00000013242	MAPKAP1	1	mitogen-activated protein kinase associated protein 1 [Source:HGNC Symbol;Acc:18752]

Organisms	Chromosome /Scaffolds	Start Position	Ending Position	Ensembl code	Short name	Strand	Annotation
Oryzias_latipes	12	6932069	6932758	ENSORLG00000003980	SURF6	1	surfeit 6 [Source:HGNC Symbol;Acc:11478]
Oryzias_latipes	12	9727776	9727941	ENSORLG00000006401	SDAD1	1	SDA1 domain containing 1 [Source:HGNC Symbol;Acc:25537]
Oryzias_latipes	13	12196172	12196324	ENSORLG00000005842	DHX36	1	DEAH (Asp-Glu-Ala-His) box polypeptide 36 [Source:HGNC Symbol;Acc:14410]
Oryzias_latipes	13	15978825	15978492	ENSORLG00000007707	UBE4A	-1	ubiquitination factor E4A [Source:HGNC Symbol;Acc:12499]
Oryzias_latipes	13	25031585	25033070	ENSORLG00000013037	SACS	1	spastic ataxia of Charlevoix-Saguenay (sacsin) [Source:HGNC Symbol;Acc:10519]
Oryzias_latipes	13	9839322	9840374	ENSORLG00000004824	WDR81	1	WD repeat domain 81 [Source:HGNC Symbol;Acc:26600]
Oryzias_latipes	14	11531348	11532671	ENSORLG00000004718	PROSER1	1	proline and serine rich 1 [Source:HGNC Symbol;Acc:20291]
Oryzias_latipes	14	11638551	11638362	ENSORLG00000004845	GCFC1	-1	GC-rich sequence DNA-binding factor 1 [Source:HGNC Symbol;Acc:13579]
Oryzias_latipes	14	17367969	17366546	ENSORLG00000009556	ZC3H12B	-1	zinc finger CCCH-type containing 12B [Source:HGNC Symbol;Acc:17407]
Oryzias_latipes	14	28795385	28795216	ENSORLG00000014405	ZW10	-1	ZW10, kinetochore associated, homolog (Drosophila) [Source:HGNC Symbol;Acc:13194]
Oryzias_latipes	15	22045429	22045286	ENSORLG00000007646	CRTAC1=(1=of=2)	-1	cartilage acidic protein 1 [Source:HGNC Symbol;Acc:14882]
Oryzias_latipes	15	24388366	24388217	ENSORLG00000010302	LMBRD1	-1	LMBR1 domain containing 1 [Source:HGNC Symbol;Acc:23038]
Oryzias_latipes	15	24713390	24714063	ENSORLG00000010603	MAP3K4	1	mitogen-activated protein kinase kinase kinase 4 [Source:HGNC Symbol;Acc:6856]
Oryzias_latipes	15	29399660	29400785	ENSORLG00000013866	INPP5F	1	inositol polyphosphate-5-phosphatase F [Source:HGNC Symbol;Acc:17054]
Oryzias_latipes	15	8927717	8929348	ENSORLG00000001816	PDZD8	1	PDZ domain containing 8 [Source:HGNC Symbol;Acc:26974]
Oryzias_latipes	16	13676353	13676225	ENSORLG00000008224	SETD2	-1	SET domain containing 2 [Source:HGNC Symbol;Acc:18420]
Oryzias_latipes	16	16874799	16874599	ENSORLG00000011742	RASIP1	-1	Ras interacting protein 1 [Source:HGNC Symbol;Acc:24716]
Oryzias_latipes	16	17719593	17719742	ENSORLG00000013063	VARS	1	valyl-tRNA synthetase [Source:HGNC Symbol;Acc:12651]
Oryzias_latipes	16	19936891	19936335	ENSORLG00000014226	MED18	-1	mediator complex subunit 18 [Source:HGNC Symbol;Acc:25944]
Oryzias_latipes	16	5748621	5750402	ENSORLG00000004142	GTDC2	1	glycosyltransferase-like domain containing 2 [Source:HGNC

Organisms	Chromosome /Scaffolds	Start Position	Ending Position	Ensembl code	Short name	Strand	Annotation
							Symbol;Acc:25902]
Oryzias_latipes	17	10584662	10582040	ENSORLG00000006746	VCPIP1	-1	valosin containing protein (p97)/p47 complex interacting protein 1 [Source:HGNC Symbol;Acc:30897]
Oryzias_latipes	17	28220772	28220917	ENSORLG00000017486	SNTG1	1	syntrophin, gamma 1 [Source:HGNC Symbol;Acc:13740]
Oryzias_latipes	17	4770658	4770847	ENSORLG00000004044	RALBP1	1	ralA binding protein 1 [Source:HGNC Symbol;Acc:9841]
Oryzias_latipes	17	4895906	4895516	ENSORLG00000004190	EPB41L3	-1	erythrocyte membrane protein band 4.1-like 3 [Source:HGNC Symbol;Acc:3380]
Oryzias_latipes	18	11250524	11250677	ENSORLG00000005132	ZDHHC21	1	zinc finger, DHHC-type containing 21 [Source:HGNC Symbol;Acc:20750]
Oryzias_latipes	19	15941013	15941164	ENSORLG00000011688	TECTB	1	tectorin beta [Source:HGNC Symbol;Acc:11721]
Oryzias_latipes	19	20451916	20451780	ENSORLG00000013774	NPLOC4	-1	nuclear protein localization 4 homolog (S. cerevisiae) [Source:HGNC Symbol;Acc:18261]
Oryzias_latipes	19	8216368	8216848	ENSORLG00000007670	WDR24	1	WD repeat domain 24 [Source:HGNC Symbol;Acc:20852]
Oryzias_latipes	19	9894312	9894157	ENSORLG00000009078	PALD1	-1	phosphatase domain containing, paladin 1 [Source:HGNC Symbol;Acc:23530]
Oryzias_latipes	20	15269857	15268841	ENSORLG00000008498	RRS1	-1	RRS1 ribosome biogenesis regulator homolog (S. cerevisiae) [Source:HGNC Symbol;Acc:17083]
Oryzias_latipes	20	15626365	15626204	ENSORLG00000008758	PAN3	-1	PAN3 poly(A) specific ribonuclease subunit homolog (S. cerevisiae) [Source:HGNC Symbol;Acc:29991]
Oryzias_latipes	20	19658802	19658489	ENSORLG00000011783	CHPF2	-1	chondroitin polymerizing factor 2 [Source:HGNC Symbol;Acc:29270]
Oryzias_latipes	20	19959910	19959756	ENSORLG00000012388	DNAJC13	-1	DnaJ (Hsp40) homolog, subfamily C, member 13 [Source:HGNC Symbol;Acc:30343]
Oryzias_latipes	21	10009015	10007406	ENSORLG00000011491	MYCBP2	-1	MYC binding protein 2, E3 ubiquitin protein ligase [Source:HGNC Symbol;Acc:23386]
Oryzias_latipes	21	22336321	22336170	ENSORLG00000016666	PRPF40A	-1	PRP40 pre-mRNA processing factor 40 homolog A (S. cerevisiae) [Source:HGNC Symbol;Acc:16463]
Oryzias_latipes	21	24051290	24051460	ENSORLG00000017359	HAT1	1	histone acetyltransferase 1 [Source:HGNC Symbol;Acc:4821]

Organisms	Chromosome /Scaffolds	Start Position	Ending Position	Ensembl code	Short name	Strand	Annotation
Oryzias_latipes	21	24127508	24126006	ENSORLG00000017400	NUP62CL	-1	nucleoporin 62kDa C-terminal like [Source:HGNC Symbol;Acc:25960]
Oryzias_latipes	21	24592238	24591830	ENSORLG00000017534	Q2WFR6_ORYLA	-1	Even-skipped homologue 2 [Source:UniProtKB/TrEMBL;Acc:Q2WFR6]
Oryzias_latipes	22	12008264	12007805	ENSORLG00000015999	BTBD7	-1	BTB (POZ) domain containing 7 [Source:HGNC Symbol;Acc:18269]
Oryzias_latipes	22	12405690	12406196	ENSORLG00000016172	KTN1	1	kinectin 1 (kinesin receptor) [Source:HGNC Symbol;Acc:6467]
Oryzias_latipes	22	14613095	14613637	ENSORLG00000017120	C14orf28	1	chromosome 14 open reading frame 28 [Source:HGNC Symbol;Acc:19834]
Oryzias_latipes	23	13312579	13312349	ENSORLG00000013384	ASUN	-1	asunder, spermatogenesis regulator homolog (Drosphila) [Source:HGNC Symbol;Acc:20174]
Oryzias_latipes	23	22793575	22794024	ENSORLG00000017184	SLC35E3	1	solute carrier family 35, member E3 [Source:HGNC Symbol;Acc:20864]
Oryzias_latipes	23	6000740	6000599	ENSORLG00000011050	SLC37A3	-1	solute carrier family 37 (glycerol-3-phosphate transporter), member 3 [Source:HGNC Symbol;Acc:20651]
Oryzias_latipes	24	17669356	17671107	ENSORLG00000017570	VRTN	1	vertebrae development homolog (pig) [Source:HGNC Symbol;Acc:20223]
Oryzias_latipes	24	19556613	19556080	ENSORLG00000017699	TFAP2D	-1	transcription factor AP-2 delta (activating enhancer binding protein 2 delta) [Source:HGNC Symbol;Acc:15581]
Oryzias_latipes	scaffold860	78177	79007	ENSORLG00000018473	PHAX	1	phosphorylated adaptor for RNA export [Source:HGNC Symbol;Acc:10241]
Oryzias_latipes	scaffold874	51941	52375	ENSORLG00000018714	USP38	1	ubiquitin specific peptidase 38 [Source:HGNC Symbol;Acc:20067]
Oryzias_latipes	ultracontig115	4231577	4236646	ENSORLG00000020551	FAT4	1	FAT tumor suppressor homolog 4 (Drosophila) [Source:HGNC Symbol;Acc:23109]
Oryzias_latipes	ultracontig25	54262	54390	ENSORLG00000020638	DMD	1	dystrophin [Source:HGNC Symbol;Acc:2928]
Oryzias_latipes	ultracontig90	1005190	1005405	ENSORLG00000018951	MTHFR	1	methylenetetrahydrofolate reductase (NAD(P)H) [Source:HGNC Symbol;Acc:7436]
Tetraodon_nigroviridis	1	10956599	10956412	ENSTNIG00000010652	HERC2	-1	HECT and RLD domain containing E3 ubiquitin protein ligase 2 [Source:HGNC Symbol;Acc:4868]
Tetraodon_nigroviridis	1	11890427	11890602	ENSTNIG00000017653	TMEM165	1	transmembrane protein 165 [Source:HGNC Symbol;Acc:30760]
Tetraodon_nigroviridis	1	16786638	16786310	ENSTNIG00000015566	PSMD1	-1	proteasome (prosome, macropain) 26S subunit, non- ATPase, 1

Organisms	Chromosome /Scaffolds	Start Position	Ending Position	Ensembl code	Short name	Strand	Annotation
							[Source:HGNC Symbol;Acc:9554]
Tetraodon_nigroviridis	1	9135156	9135350	ENSTNIG00000016852	TAF7	1	TAF7 RNA polymerase II, TATA box binding protein (TBP)-associated factor, 55kDa [Source:HGNC Symbol;Acc:11541]
Tetraodon_nigroviridis	2	10050233	10050449	ENSTNIG00000014978	TMTC4	1	transmembrane and tetratricopeptide repeat containing 4 [Source:HGNC Symbol;Acc:25904]
Tetraodon_nigroviridis	2	11239859	11240113	ENSTNIG00000013871	TXNDC9	1	thioredoxin domain containing 9 [Source:HGNC Symbol;Acc:24110]
Tetraodon_nigroviridis	2	13630568	13631822	ENSTNIG00000016947	SP3=(1=of=2)	1	Sp3 transcription factor [Source:HGNC Symbol;Acc:11208]
Tetraodon_nigroviridis	2	13912729	13912299	ENSTNIG00000016925	UBR3=(2=of=2)	-1	ubiquitin protein ligase E3 component n-recognin 3 (putative) [Source:HGNC Symbol;Acc:30467]
Tetraodon_nigroviridis	2	14121860	14121672	ENSTNIG00000016508	TTC21B	-1	tetratricopeptide repeat domain 21B [Source:HGNC Symbol;Acc:25660]
Tetraodon_nigroviridis	2	1771535	1771798	ENSTNIG00000010090	MSL1	1	male-specific lethal 1 homolog (Drosophila) [Source:HGNC Symbol;Acc:27905]
Tetraodon_nigroviridis	2	5947345	5947183	ENSTNIG00000012906	MLST8	-1	MTOR associated protein, LST8 homolog (S. cerevisiae) [Source:HGNC Symbol;Acc:24825]
Tetraodon_nigroviridis	2	6461364	6461546	ENSTNIG00000012867	SLC38A10	1	solute carrier family 38, member 10 [Source:HGNC Symbol;Acc:28237]
Tetraodon_nigroviridis	2	9149046	9149202	ENSTNIG00000011538	RIF1	1	RAP1 interacting factor homolog (yeast) [Source:HGNC Symbol;Acc:23207]
Tetraodon_nigroviridis	2	9626068	9626286	ENSTNIG00000005285	RPL8	1	ribosomal protein L8 [Source:HGNC Symbol;Acc:10368]
Tetraodon_nigroviridis	2	9641348	9641130	ENSTNIG00000014968	USP9X	-1	ubiquitin specific peptidase 9, X-linked [Source:HGNC Symbol;Acc:12632]
Tetraodon_nigroviridis	3	108213	108348	ENSTNIG00000010613	PWP2	1	PWP2 periodic tryptophan protein homolog (yeast) [Source:HGNC Symbol;Acc:9711]
Tetraodon_nigroviridis	3	11617797	11618019	ENSTNIG00000001019	ATP13A1=(1=of=2)	1	ATPase type 13A1 [Source:HGNC Symbol;Acc:24215]
Tetraodon_nigroviridis	4	2449628	2449221	ENSTNIG00000009041	KIAA1432	-1	KIAA1432 [Source:HGNC Symbol;Acc:17686]
Tetraodon_nigroviridis	4	8079218	8077197	ENSTNIG00000018740	TRIM32	-1	tripartite motif containing 32 [Source:HGNC Symbol;Acc:16380]
Tetraodon_nigroviridis	5	10408483	10411190	ENSTNIG00000009552	UACA	1	uveal autoantigen with coiled-coil domains and ankyrin repeats [Source:HGNC Symbol;Acc:15947]

Organisms	Chromosome /Scaffolds	Start Position	Ending Position	Ensembl code	Short name	Strand	Annotation
Tetraodon_nigroviridis	5	4306400	4306543	ENSTNIG00000013807	EIF3M	1	eukaryotic translation initiation factor 3, subunit M [Source:HGNC Symbol;Acc:24460]
Tetraodon_nigroviridis	5	5173813	5173627	ENSTNIG00000009298	STRC	-1	stereocilin [Source:HGNC Symbol;Acc:16035]
Tetraodon_nigroviridis	5	6333994	6333755	ENSTNIG00000009364	CTR9	-1	Ctr9, Paf1/RNA polymerase II complex component, homolog (S. cerevisiae) [Source:HGNC Symbol;Acc:16850]
Tetraodon_nigroviridis	6	5001368	5001534	ENSTNIG00000012785	UBE3C	1	ubiquitin protein ligase E3C [Source:HGNC Symbol;Acc:16803]
Tetraodon_nigroviridis	7	5939818	5939611	ENSTNIG00000011788	TAF1L	-1	TAF1 RNA polymerase II, TATA box binding protein (TBP)-associated factor, 210kDa-like [Source:HGNC Symbol;Acc:18056]
Tetraodon_nigroviridis	8	8678765	8679146	ENSTNIG00000000738	MBOAT7	1	membrane bound O-acyltransferase domain containing 7 [Source:HGNC Symbol;Acc:15505]
Tetraodon_nigroviridis	9	10438933	10436663	ENSTNIG00000006517	VPS13D	-1	vacuolar protein sorting 13 homolog D (S. cerevisiae) [Source:HGNC Symbol;Acc:23595]
Tetraodon_nigroviridis	9	1435959	1435825	ENSTNIG00000011975	PRDM16	-1	PR domain containing 16 [Source:HGNC Symbol;Acc:14000]
Tetraodon_nigroviridis	9	2299092	2298186	ENSTNIG00000015174	CASZ1	-1	castor zinc finger 1 [Source:HGNC Symbol;Acc:26002]
Tetraodon_nigroviridis	9	3228741	3228571	ENSTNIG00000001264	RPL10A	-1	ribosomal protein L10a [Source:HGNC Symbol;Acc:10299]
Tetraodon_nigroviridis	9	4099112	4097670	ENSTNIG00000015053	MFSD5	-1	major facilitator superfamily domain containing 5 [Source:HGNC Symbol;Acc:28156]
Tetraodon_nigroviridis	9	4711544	4711884	ENSTNIG00000015029	LGR6	1	leucine-rich repeat containing G protein-coupled receptor 6 [Source:HGNC Symbol;Acc:19719]
Tetraodon_nigroviridis	9	5721943	5721791	ENSTNIG00000017873	PRPF6	-1	PRP6 pre-mRNA processing factor 6 homolog (S. cerevisiae) [Source:HGNC Symbol;Acc:15860]
Tetraodon_nigroviridis	10	11026450	11028852	ENSTNIG00000019281	MLL=(2=of=2)	1	myeloid/lymphoid or mixed-lineage leukemia (trithorax homolog, Drosophila) [Source:HGNC Symbol;Acc:7132]
Tetraodon_nigroviridis	10	2456927	2456692	ENSTNIG00000005645	KIAA1109=(2=of=2)	-1	KIAA1109 [Source:HGNC Symbol;Acc:26953]
Tetraodon_nigroviridis	10	4326409	4326970	ENSTNIG00000000917	ZBTB42	1	zinc finger and BTB domain containing 42 [Source:HGNC Symbol;Acc:32550]
Tetraodon_nigroviridis	10	5462041	5462250	ENSTNIG00000017381	EXOC5	1	exocyst complex component 5 [Source:HGNC Symbol;Acc:10696]

Organisms	Chromosome /Scaffolds	Start Position	Ending Position	Ensembl code	Short name	Strand	Annotation
Tetraodon_nigroviridis	10	5837504	5837191	ENSTNIG00000017365	UNC79	-1	unc-79 homolog (C. elegans) [Source:HGNC Symbol;Acc:19966]
Tetraodon_nigroviridis	11	3046273	3046090	ENSTNIG00000007070	EYA2	-1	eyes absent homolog 2 (Drosophila) [Source:HGNC Symbol;Acc:3520]
Tetraodon_nigroviridis	11	6646765	6646626	ENSTNIG00000014655	SSU72	-1	SSU72 RNA polymerase II CTD phosphatase homolog (S. cerevisiae) [Source:HGNC Symbol;Acc:25016]
Tetraodon_nigroviridis	12	11834359	11834181	ENSTNIG00000011475	MLEC	-1	malectin [Source:HGNC Symbol;Acc:28973]
Tetraodon_nigroviridis	13	5229998	5230908	ENSTNIG00000012152	BHLHE41	1	basic helix-loop-helix family, member e41 [Source:HGNC Symbol;Acc:16617]
Tetraodon_nigroviridis	13	5945359	5946642	ENSTNIG00000011377	FJX1	1	four jointed box 1 (Drosophila) [Source:HGNC Symbol;Acc:17166]
Tetraodon_nigroviridis	14	1552621	1552753	ENSTNIG00000019156	TTC13	1	tetratricopeptide repeat domain 13 [Source:HGNC Symbol;Acc:26204]
Tetraodon_nigroviridis	14	4374886	4374628	ENSTNIG00000010757	LPGAT1	-1	lysophosphatidylglycerol acyltransferase 1 [Source:HGNC Symbol;Acc:28985]
Tetraodon_nigroviridis	14	5003764	5004693	ENSTNIG00000016391	SLC35B2	1	solute carrier family 35, member B2 [Source:HGNC Symbol;Acc:16872]
Tetraodon_nigroviridis	16	3836777	3836983	ENSTNIG00000016222	KIAA0100	1	KIAA0100 [Source:HGNC Symbol;Acc:28960]
Tetraodon_nigroviridis	16	6376552	6377063	ENSTNIG00000018994	GPR4	1	G protein-coupled receptor 4 [Source:HGNC Symbol;Acc:4497]
Tetraodon_nigroviridis	17	1066767	1066532	ENSTNIG00000013095	SIRT1	-1	sirtuin 1 [Source:HGNC Symbol;Acc:14929]
Tetraodon_nigroviridis	17	3438200	3440765	ENSTNIG00000016603	MSH6	1	mutS homolog 6 (E. coli) [Source:HGNC Symbol;Acc:7329]
Tetraodon_nigroviridis	17	610910	610401	ENSTNIG00000002975	PHF14	-1	PHD finger protein 14 [Source:HGNC Symbol;Acc:22203]
Tetraodon_nigroviridis	17	6600380	6600160	ENSTNIG00000008621	BFSP1	-1	beaded filament structural protein 1, filensin [Source:HGNC Symbol;Acc:1040]
Tetraodon_nigroviridis	17	7919883	7920472	ENSTNIG00000013381	TJAP1	1	tight junction associated protein 1 (peripheral) [Source:HGNC Symbol;Acc:17949]
Tetraodon_nigroviridis	17	909512	909682	ENSTNIG00000013082	SORBS1	1	sorbin and SH3 domain containing 1 [Source:HGNC Symbol;Acc:14565]
Tetraodon_nigroviridis	18	1479251	1479126	ENSTNIG00000014137	P4HB	-1	prolyl 4-hydroxylase, beta polypeptide [Source:HGNC Symbol;Acc:8548]
Tetraodon_nigroviridis	18	2859535	2859743	ENSTNIG00000010580	SHOC2	1	soc-2 suppressor of clear homolog (C. elegans) [Source:HGNC Symbol;Acc:15454]
Tetraodon_nigroviridis	18	9516502	9517205	ENSTNIG00000008275	ALKBH5	1	alkB, alkylation repair homolog 5 (E. coli) [Source:HGNC

Organisms	Chromosome /Scaffolds	Start Position	Ending Position	Ensembl code	Short name	Strand	Annotation
							Symbol;Acc:25996]
Tetraodon_nigroviridis	19	1658177	1654696	ENSTNIG00000012690	GOLGB1	-1	golgin B1 [Source:HGNC Symbol;Acc:4429]
Tetraodon_nigroviridis	21	5083783	5084055	ENSTNIG00000014017	KCNK9	1	potassium channel, subfamily K, member 9 [Source:HGNC Symbol;Acc:6283]
Tetraodon_nigroviridis	2_random	187782	185800	ENSTNIG00000004334	MED1	-1	mediator complex subunit 1 [Source:HGNC Symbol;Acc:9234]
Tetraodon_nigroviridis	Un_random	13372107	13371920	ENSTNIG00000003154	SETD5	-1	SET domain containing 5 [Source:HGNC Symbol;Acc:25566]
Tetraodon_nigroviridis	Un_random	16701726	16701866	ENSTNIG00000006558	SLMAP	1	sarcolemma associated protein [Source:HGNC Symbol;Acc:16643]
Tetraodon_nigroviridis	Un_random	20230713	20230553	ENSTNIG00000017616	RFWD2	-1	ring finger and WD repeat domain 2, E3 ubiquitin protein ligase [Source:HGNC Symbol;Acc:17440]
Tetraodon_nigroviridis	Un_random	2061822	2061662	ENSTNIG00000005051	ZNF341	-1	zinc finger protein 341 [Source:HGNC Symbol;Acc:15992]
Tetraodon_nigroviridis	Un_random	2936515	2937009	ENSTNIG00000010894	UBIAD1	1	UbiA prenyltransferase domain containing 1 [Source:HGNC Symbol;Acc:30791]
Tetraodon_nigroviridis	Un_random	30449827	30449639	ENSTNIG00000003399	EFTUD1	-1	elongation factor Tu GTP binding domain containing 1 [Source:HGNC Symbol;Acc:25789]
Tetraodon_nigroviridis	Un_random	31223845	31224002	ENSTNIG00000003092	POLD2	1	polymerase (DNA directed), delta 2, accessory subunit [Source:HGNC Symbol;Acc:9176]
Tetraodon_nigroviridis	Un_random	32021626	32021883	ENSTNIG00000005713	SF3A1	1	splicing factor 3a, subunit 1, 120kDa [Source:HGNC Symbol;Acc:10765]
Tetraodon_nigroviridis	Un_random	4429994	4430231	ENSTNIG00000011151	NAA60	1	N(alpha)-acetyltransferase 60, NatF catalytic subunit [Source:HGNC Symbol;Acc:25875]
Tetraodon_nigroviridis	Un_random	67198066	67197931	ENSTNIG00000004176	CCM2	-1	cerebral cavernous malformation 2 [Source:HGNC Symbol;Acc:21708]
Tetraodon_nigroviridis	Un_random	67565861	67565588	ENSTNIG00000004207	TTL	-1	tubulin tyrosine ligase [Source:HGNC Symbol;Acc:21586]
Tetraodon_nigroviridis	Un_random	71604006	71603646	ENSTNIG00000004735	C19orf6	-1	chromosome 19 open reading frame 6 [Source:HGNC Symbol;Acc:17039]
Tetraodon_nigroviridis	Un_random	78084470	78085196	ENSTNIG00000005441	DICER1	1	dicer 1, ribonuclease type III [Source:HGNC Symbol;Acc:17098]
Tetraodon_nigroviridis	Un_random	95094358	95093518	ENSTNIG00000017088	PWWP2B	-1	PWWP domain containing 2B [Source:HGNC Symbol;Acc:25150]
Tetraodon_nigroviridis	Un_random	9704099	9703965	ENSTNIG00000007140	RPL36	-1	ribosomal protein L36 [Source:HGNC Symbol;Acc:13631]

Supplementary Table S8. p-distance between the unknown query samples and the other sinipercids in the database based on 500 nuclear loci. The underlined samples were used for reconstructing species tree

	Unknown samples					
	839_3		839_6		938_1	
Sample compared / distance	*Sini. kneri* 839_4	0.00054	*Sini. kneri* 839_4	0.00079	*Sini. chuatsi* 943_1	0.00035
	Sini. kneri 839_5	0.00061	*Sini. kneri* 839_3	0.00079	*Sini. chuatsi* 942_1	0.00038
	Sini. kneri 839_1	0.00065	*Sini. kneri* 839_5	0.00085	*Sini. chuatsi* 955_1	0.00046
	Sini. kneri 839_6	0.00079	*Sini. kneri* 839_1	0.00087	*Sini. chuatsi* 951_1	0.00070
	Sini. chuatsi 938_1	0.00357	*Sini. chuatsi* 938_1	0.00402	*Sini. kneri* 839_3	0.00357
	Sini. chuatsi 951_1	0.00381	*Sini. chuatsi* 943_1	0.00423	*Sini. kneri* 839_4	0.00364
	Sini. chuatsi 943_1	0.00393	*Sini. chuatsi* 942_1	0.00426	*Sini. kneri* 839_1	0.00370
	Sini. chuatsi 942_1	0.00396	*Sini. chuatsi* 955_1	0.00432	*Sini. kneri* 839_5	0.00389
	Sini. chuatsi 955_1	0.00400	*Sini. chuatsi* 951_1	0.00432	*Sini. kneri* 839_6	0.00402
	Sini. undulata 946_2	0.00551	*Sini. undulata* 946_2	0.00595	*Sini. undulata* 946_2	0.00579
	Sini. undulata 946	0.00556	*Sini. undulata* 946	0.00599	*Sini. undulata* 946	0.00582
	Sini. obscura 934_1	0.00608	*Sini. obscura* 934_1	0.00649	*Sini. obscura* 934_1	0.00630
	Sini. roulei 961_2	0.00664	*Sini. obscura* 939-1_2	0.00720	*Sini. scherzeri* 944_4	0.00696
	Sini. roulei 961_3	0.00671	*Sini. roulei* 961_2	0.00720	*Sini. roulei* 961_2	0.00697
	Sini. scherzeri 944_4	0.00683	*Sini. roulei* 947	0.00721	*Sini. roulei* 947	0.00698
	Sini. obscura 939-1_2	0.00685	*Sini. scherzeri* 944_4	0.00724	*Sini. roulei* 961_3	0.00703
	Sini. roulei 947	0.00696	*Sini. roulei* 961_3	0.00728	*Sini. obscura* 939-1_2	0.00714
	Sini. obscura 937_2	0.00718	*Sini. obscura* 937_2	0.00761	*Sini. obscura* 937_2	0.00750
	Sini. obscura 934_2	0.00755	*Sini. obscura* 934_2	0.00777	*Sini. obscura* 934_2	0.00768
	Sini. obscura 937_3	0.00764	*Sini. obscura* 937_3	0.00793	*Sini. obscura* 937_3	0.00775
	C.whiteheadi 831_1	0.03778	*C. whiteheadi* 940_1	0.03867	*Sini. whiteheadi* 831_1	0.03764
	C.whiteheadi 940_1	0.03790	*C. whiteheadi* 831_1	0.03880	*Sini. whiteheadi* 940_1	0.03797
	C.whiteheadi 940_2	0.03836	*C. whiteheadi* 940_2	0.03889	*Sini. whiteheadi* 940_2	0.03814
	C.whiteheadi 958_1	0.03836	*C. whiteheadi* 958_1	0.03896	*Sini. whiteheadi* 958_1	0.03824
	C.whiteheadi 945_3	0.03877	*C. whiteheadi* 945_3	0.03947	*Sini. whiteheadi* 945_3	0.03863

Supplementary Table S9. Results for species delimitation on unknown sample 893_3 (*Sini. kneri*), 839_6 (*Sini. kneri*) and 938_1 (*Sini. chuatsi*) using BFD*

Model	Marginal likelihood	2lnBF
Lumping 839_3 and *Sini. kneri*	-1575.80	20.62
Splitting 839_3 and *Sini. kneri*	-1586.11	
Lumping 839_6 and *Sini. kneri*	-1287.70	22.02
Splitting 839_6 and *Sini. kneri*	-1298.71	
Lumping 938_1 and *Sini. chautsi*	-1287.70	6.46
Splitting 938_1 and *Sini. chautsi*	-1290.93	

Supplementary Table S10. P-distance between the unknown query samples and the samples in the database based on COI sequences

	Unknown sample					
	Sini. kneri 839_3		*Sini. kneri* 839_6		*Sini. chuatsi* 938_1	
Sample compared / distance	*Sini. kneri* 839_4	0.08675	*Sini. chuatsi* 943_1	0.14369	*Sini. chuatsi* 942_1	0.00389
	Sini. chuatsi 938_1	0.11916	*Sini. chuatsi* 942_1	0.14514	*Sini. chuatsi* 943_1	0.00877
	Sini. chuatsi 943_1	0.12397	*Sini. chuatsi* 938_1	0.14949	*Sini. scherzeri* 944_4	0.04104
	Sini. chuatsi 942_1	0.13049	*Sini. kneri* 839_4	0.17101	*Sini. undulata* 946	0.05527
	Sini. scherzeri 944_4	0.13333	*Sini. scherzeri* 944_4	0.17707	*Sini. undulata* 946_2	0.05799
	Sini. roulei 961_3	0.13675	*Sini. kneri* 839_1	0.17971	*Sini. roulei* 961_3	0.06557
	Sini. obscura 937_2	0.13962	*Sini. undulata* 946	0.19013	*Sini. roulei* 947	0.06801
	Sini. roulei 947	0.14713	*Sini. undulata* 946_2	0.19158	*Sini. roulei* 961_2	0.07749
	Sini. undulata 946	0.15115	*Sini. kneri* 839_3	0.20029	*Sini. kneri* 839_4	0.08394
	Sini. roulei 961_2	0.15304	*Sini. roulei* 961_3	0.20174	*Sini. kneri* 839_3	0.11916
	Sini. undulata 946_2	0.16889	*Sini. roulei* 961_2	0.20319	*Sini. chuatsi* 951_1	0.13043
	C.whiteheadi 831_1	0.19847	*Sini. roulei* 947	0.20464	*Sini. obscura* 934_1	0.14446
	Sini. kneri 839_6	0.20029	*Sini. chuatsi* 951_1	0.25526	*Sini. chuatsi* 955-1	0.14867
	C.whiteheadi 940_1	0.20665	*C.whiteheadi* 831-1	0.26232	*Sini. kneri* 839_6	0.14949
	Sini. obscura 934_1	0.21031	*Sini. obscura* 934_1	0.26232	*C.whiteheadi* 940_1	0.17197
	C.whiteheadi 940_2	0.22184	*Sini. chuatsi* 955-1	0.27027	*C. whiteheadi* 940-2	0.17317
	Sini. chuatsi 951_1	0.22441	*C.whiteheadi* 940-2	0.27246	*Sini. kneri* 839_1	0.17418
	Sini. chuatsi 955_1	0.23753	*Sini. obscura* 937_2	0.27536	*C. whiteheadi* 831-1	0.17528
	Sini. kneri 839_1	0.24942	*C.whiteheadi* 940_1	0.27541	*Sini. obscura* 937_2	0.18856
	C.whiteheadi 945_3	0.25145	*C.whiteheadi* 958_1	0.28529	*Sini. obscura* 937_3	0.20448
	Sini. obscura 937_3	0.26720	*Sini. obscura* 937_3	0.29279	*C.whiteheadi* 958_1	0.21739
	C. whiteheadi 958_1	0.27067	*Sini. whiteheadi* 945_3	0.32733	*C.whiteheadi* 945_3	0.22965
	Sini. obscura 934_2	0.31178	*Sini. obscura* 934_2	0.39039	*Sini. obscura* 934_2	0.27155
	Sini. obscura 939_1_2	0.39752	*Sini. obscura* 939_1_2	0.44733	*Sini. obscura* 939_1_2	0.35624
	Sini. kneri 839_5	0.40385	*Sini. kneri* 839_5	0.45496	*Sini. kneri* 839_5	0.35624

Supplementary Table S11. P-distance between the unknown query 839_3 (*Siniperca kneri*) and the sinipercids in the database. Conspecifics of *Sini. kneri* were excluded from the database

Sample compared	p-distance
Siniperca chuatsi 938_1	0.00357
Siniperca chuatsi 951_1	0.00381
Siniperca chuatsi 943_1	0.00393
Siniperca chuatsi 942_1	0.00396
Siniperca chuatsi 955_1	0.00400
Siniperca undulata 946_2	0.00551
Siniperca undulata 946	0.00556
Siniperca obscura 934_1	0.00608
Siniperca roulei 961_2	0.00664
Siniperca roulei 961_3	0.00671
Siniperca scherzeri 944_4	0.00683
Siniperca obscura 939-1_2	0.00685
Siniperca roulei 947	0.00696
Siniperca obscura 937_2	0.00718
Siniperca obscura 934_2	0.00755
Siniperca obscura 937_3	0.00764
Coreoperca whiteheadi 831_1	0.03778
Coreoperca whiteheadi 940_1	0.03790
Coreoperca whiteheadi 940_2	0.03836
Coreoperca whiteheadi 958_1	0.03836
Coreoperca whiteheadi 945_3	0.03877

Supplementary Table S12. Twenty markers with least missing data and longest alignment

Model species	Chromosomal position	Ensembl gene name	Short name	gene description
Danio rerio	1:28199081:28201810	ENSDARG00000060620	lig4	ligase IV, DNA, ATP-dependent [Source:ZFIN;Acc:ZDB- GENE-070820-10]
Danio rerio	10:33603205:33606281	ENSDARG00000043285	znf295	zinc finger protein 295 [Source:ZFIN;Acc:ZDB-GENE- 050411-7]
Danio rerio	14:33713005:33715226	ENSDARG00000055040	nkrf	NF-kappa B repressing factor [Source:ZFIN;Acc:ZDB- GENE-030616-73]
Danio rerio	20:15058321:15055829	ENSDARG00000057583	prrc2c	proline-rich coiled-coil 2C [Source:ZFIN;Acc:ZDB- GENE-081104-156]
Danio rerio	20:4037479:4035425	ENSDARG00000063211	exoc8	exocyst complex component 8 [Source:ZFIN;Acc:ZDB- GENE-070410-60]
Danio rerio	20:50273765:50271624	ENSDARG00000026811	extl3	exostoses (multiple)-like 3 [Source:ZFIN;Acc:ZDB- GENE-041124-2]
Danio rerio	21:18734269:18731416	ENSDARG00000079104	mfhas1	malignant fibrous histiocytoma amplified sequence 1 [Source:ZFIN;Acc:ZDB-GENE-080917-20]
Danio rerio	7:61639537:61643442	ENSDARG00000077162	—	—
Gasterosteus aculeatus	groupVI:6368659:6364893	ENSGACG00000005370	GOLGA4	golgin A4 [Source:HGNC Symbol;Acc:4427]
Gasterosteus aculeatus	groupX:1864277:1870053	ENSGACG00000002352	HIVEP1	human immunodeficiency virus type I enhancer binding protein 1 [Source:HGNC Symbol;Acc:4920]
Gasterosteus aculeatus	groupXV:12188570:12191611	ENSGACG00000012223	—	—
Oryzias latipes	1:24751546:24754136	ENSORLG00000008328	DCHS2	dachsous 2 (Drosophila) [Source:HGNC Symbol;Acc:23111]
Oryzias latipes	10:14233039:14235746	ENSORLG00000006198	FRMPD3	FERM and PDZ domain containing 3 [Source:HGNC Symbol;Acc:29382]
Oryzias latipes	17:10584662:10582040	ENSORLG00000006746	VCPIP1	valosin containing protein (p97)/p47 complex interacting protein 1 [Source:HGNC Symbol;Acc:30897]
Oryzias latipes	ultracontig115:4231577:4236646	ENSORLG00000020551	FAT4	FAT tumor suppressor homolog 4 (Drosophila) [Source:HGNC Symbol;Acc:23109]
Tetraodon nigroviridis	10:11026450:11028852	ENSTNIG00000019281	MLL (2 of 2)	myeloid/lymphoid or mixed-lineage leukemia (trithorax homolog, Drosophila) [Source:HGNC Symbol;Acc:7132]
Tetraodon nigroviridis	17:3438200:3440765	ENSTNIG00000016603	MSH6	mutS homolog 6 (E. coli) [Source:HGNC Symbol;Acc:7329]
Tetraodon nigroviridis	4:8079218:8077197	ENSTNIG00000018740	TRIM32	tripartite motif containing 32 [Source:HGNC Symbol;Acc:16380]
Tetraodon nigroviridis	5:10408483:10411190	ENSTNIG00000009552	UACA	uveal autoantigen with coiled-coil domains and ankyrin repeats [Source:HGNC Symbol;Acc:15947]
Tetraodon nigroviridis	9:10438933:10436663	ENSTNIG00000006517	VPS13D	vacuolar protein sorting 13 homolog D (S. cerevisiae) [Source:HGNC Symbol;Acc:23595]

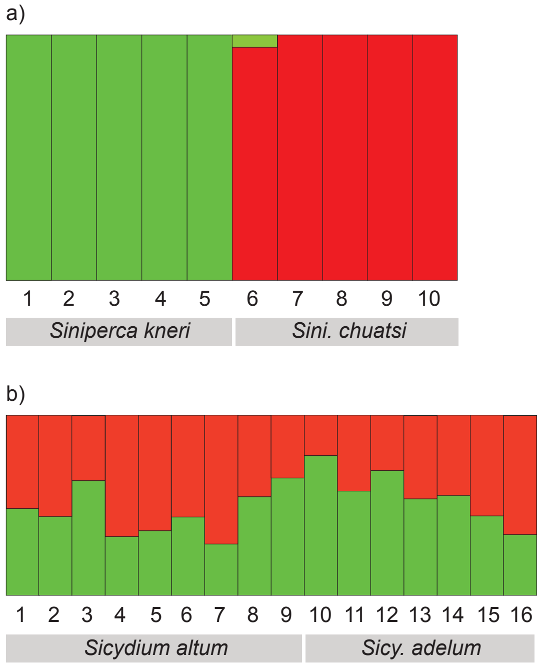

Supplementary Figure S1. Results of Structure analyses in *Siniperca kneri* (a. 1-5) and *Sini. chuatsi* (a. 6-10), and in *Sicydium altum* (b. 1-9) and *Sicy. adelum* (b. 10-16).

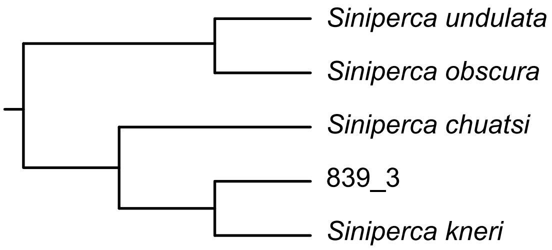

Supplementary Figure S2. The species tree reconstructed for multilocus DNA barcoding on sample 839_3. Four closely related species in addition to the unknown (839_3) were used to infer the species tree.

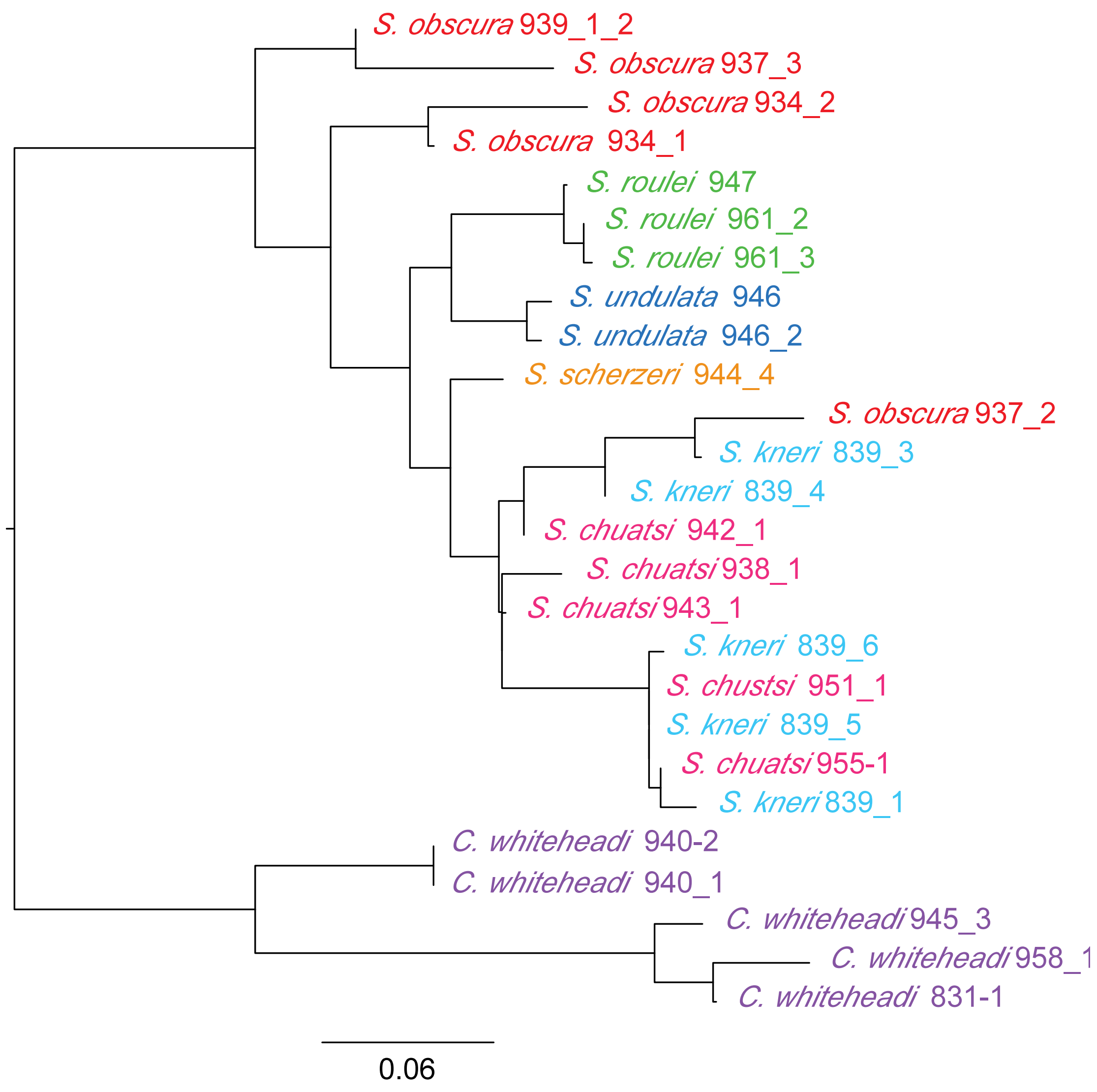

Supplementary Figure S3. Phylogeny of the sinipercids based on COI sequences. Different colors indicate different species.

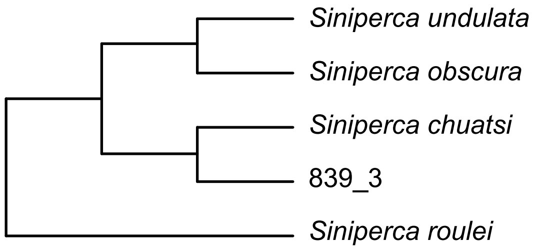

Supplementary Figure S4. The species tree reconstructed for multilocus DNA barcoding on sample 839_3. All conspecifics of 839_3 were excluded from the analysis. Four closely related species in addition to the unknown (839_3) were used to infer the species tree.
