# Supplementary material for: Multilocus DNA barcoding – Species Identification with Multilocus Data
Source: Sci Rep. 2017 Nov 30;7:16601. doi: 10.1038/s41598-017-16920-2 (PMC5709489; doi:10.1038/s41598-017-16920-2)
Supplement: Supplementary file 2 — Perl scripts [file 41598_2017_16920_MOESM2_ESM.pdf]

Multilocus DNA barcoding – Species Identification with Multilocus Data

Junning Liu, Jiamei Jiang, Shuli Song, Luke Tornabene, Ryan Chabarria, Gavin J P Naylor, Chenhong Li

Perl scripts:

**picktaxagene.pl**	pick out individuals without missing data
**distoutlier.pl**	sort loci by their average p-distance
**samplegene.pl**	different number of loci randomly and concatenated them
**gapdis.pl**	calculate p-distance among individuals
**ID_correct_rate.pl**	calculate the rate of successful identification (with the pdistance.pm module)
**pdistance.pl**	calculate the p-distance between query and other individuals in database
**concatnexus.pl**	read the fasta sequences from each file and concatenate them into a nexus file

```
#!/usr/bin/perl -w

##################################################################################
#
# picktaxagene.pl
#
# select fasta sequences for a given list of taxa from each file and output new fasta file if the
# number of picked taxa is greater than a given value.
#
# Input: the list of species name and the directory storing the fasta files and a preset minimum
# value for the number of taxa.
#
# Output: a directory storing the new fasta files
#
# Written by
#                       Chenhong Li
#                       Shanghai Ocean University, China.
#                       Created on Oct 2015
#                       Last modified at
#
##################################################################################

use warnings;

use Getopt::Long;      # include the module for input

my $dir = "siniperca_all";; #variable for store the directory
my $outdir = $dir . "selected"; #variable for store the output directory
my $specieslist = "Sin_chu_CL938_1 Sin_chu_CL942_2"; #variable for store the list of species
                         name
#my $minnumseq = 10; #the minimum number of sequences for keeping a locus

my $opt = GetOptions( 'dir:s', \$dir,
                           'outdir:s', \$outdir,
                           'min:s', \$minnumseq,
                           'specieslist:s', \$specieslist); #set command line options

`mkdir $outdir`; #make output folder

#print "$specieslist\n";
my @list = split/\s/, $specieslist;
@list = sort @list;
#print "@list\n";
```

```
opendir (DIR, $dir) or die $!; #open folder containing all fasta data file
my ($IN_FILE, $OUT_FILE, $infilename, $outfile);


while (my $file = readdir(DIR)) { #read all fasta files under the folder

    next if ($file =~ /^\./); #skip files beginning with .
    $infilename = "$dir/$file"; #get the name of fasta file
    open ($IN_FILE, "<$infilename") or die "Can't open the    file!!!";
    $outfile = "$outdir/$file";
    open ($OUT_FILE, ">$outfile") or die "Can't open the output file!!!";

    my $countseq=0;
    while (my $line = readline ($IN_FILE)) {
        chomp $line;
        if (my ($speciesname) = $line =~ /^>(\S+)/) { # if we find >
            if (grep{$_ eq $speciesname} @list) {#if speciesname found in list
                my $nextline = readline ($IN_FILE); #read the sequence in the next line
                chop $nextline;
                $countseq ++;
                print $OUT_FILE ">$speciesname\n$nextline\n";
                #print ">$speciesname\n$nextline\n";

            }
        }
    }
    print "$countseq\t$minnumseq\n";
    `rm $outfile` if ($countseq < $minnumseq); #remove the file if the number of sequences is
                    less than preset minimum

    close ($IN_FILE) or die "Can't close the input file!!!";
    close ($OUT_FILE) or die "Can't close the outfile!!!";

}

closedir (DIR);
```

```
#!/usr/bin/perl -w

#####################################################################################
#
# distoutlier.pl
#
# Compare distance among different taxa for different genes to spot the gene with bad data or bad
# alignment. First, calculate the ratio of each pairwise distance to the average pairwise distance.
# Then, calculate the average of these ratio for all genes. Last, compare the ratios calculated on
# each gene to the average value of all genes, and calculate a chi-square test values.
#
#
# Input: the directory storing the fasta files of the genes.
#
# Output: result file list the gene name and chi-square values for each gene
#
# Written by
#			Chenhong Li
#			Shanghai Ocean University, China
#			Created on Jan 2015
#			Last modified on
#
#####################################################################################

use warnings;

use Getopt::Long;	# include the module for input
use List::Util qw< sum >;
use Statistics::Distributions qw< chisqrprob >;

my $dir = "test"; #variable for store the directory
my $outfile = "out.txt"; #variable for store the directory

my $opt = GetOptions( 'dir:s', \$dir,
				'outfile:s', \$outfile); #set command line options

my ($OUT_FILE); #define output file
open ($OUT_FILE, ">$outfile") or die "Can't open the	file!!!";

opendir (DIR, $dir) or die $!; #open folder containing all fasta data file
my ($IN_FILE, $infilename);
my %dist;
my @distancekeys;
my @files;
```

```
my $genecount = 0;

while (my $file = readdir(DIR)) { #read all fasta files under the folder
	next if ($file =~ /^\./); #skip files beginning with .
	$infilename = "$dir/$file"; #get the name of fasta file
	open ($IN_FILE, "<$infilename") or die "Can't open the 	file!!!";

	my %sequence;
	while (my $line = readline ($IN_FILE)) {
		chop $line; #use chop bc there are maybe hidden characters in dos format files
		if (my ($speciesname) = $line =~ /^>(\S+)/) { # if we find >
			my $nextline = readline ($IN_FILE); #read the sequence in the next line
			chop $nextline;
			$sequence{$speciesname} = $nextline; #assign the sequence to the hash
		}
	}

# make a new hash that could be used for sequence pairwise comparison
	#assign the sequence hash into an anonymous hash keyed by both a sequencial number
	my @keys1 = keys %sequence; #get the index of the hash
	my $numoftaxa = scalar @keys1; #get the number of entry in the hash
	my %newseq;
	for (my $n = 0 ; $n < $numoftaxa ; ++$n) {
		$newseq{$n} = {taxon => $keys1[$n],
					sequence => $sequence{$keys1[$n]}};
	}

# Iterate through all pairs of sequences
	my @distkeys;
	my $totaldist = 0;
	my $numofdist; #count for number of pairwise distance
	for (my $k = 0 ; $k < $numoftaxa -1 ; ++$k) {
		for (my $i = ($k + 1) ; $i < $numoftaxa ; ++$i) {

			my $dist = 1 - matching_percentage($newseq{$k}->{sequence},
$newseq{$i}->{sequence});# Calculate and the matching percentage
			my $distkey = "$newseq{$k}->{taxon}" . "_" . "$newseq{$i}->{taxon}"; #make
the key for pairwise comparison
			push @distkeys, $distkey;
			$dist{$file} -> {$distkey} -> {distance} = $dist; #assign the distance to 	hash
			if ($dist != 1) {#if dist not equal to one
				$totaldist += $dist; #calculate the total distance
				$numofdist ++;
			}
```

```
		}
	}
	@distancekeys = @distkeys;
	push @files, $file;

	#calculate distance ratio of each pairwise comparison to average distance and add it to the
hash
	foreach my $distkey (@distkeys) {
		my $ratio = ($dist{$file}->{$distkey}->{distance} * $numofdist) / $totaldist; #calculate
ratio
		$ratio = 1 if ($dist{$file}->{$distkey}->{distance} == 1); #assign one to the ratio if
there were no overlap between two taxa
		$dist{$file} -> {$distkey} -> {ratio} = $ratio;
	}
	#print $OUT_FILE "$file\t$len\t$result\n";

	close ($IN_FILE) or die "Can't close the input file!!!";
	$genecount ++; #count the numofgenes
}

my %distmean;
foreach my $distkey (@distancekeys) {
	my $ratio = 0;
	foreach my $file (@files) {
		$ratio = $ratio + $dist{$file}->{$distkey}->{ratio};
	}
	my $avrratio = $ratio / $genecount;
	$distmean{$distkey} = $avrratio;
}

foreach my $file (@files) {
	my @observed;
	my @predicted;
	foreach my $distkey (@distancekeys) {
		push @observed, $dist{$file}->{$distkey}->{ratio};
		push @predicted, $distmean{$distkey};
	}
	my ($prob, $chisum) = chi_squared_test (\@observed, \@predicted);
	#print "$file\t$prob\n";
	#print "@observed\n@predicted\n";
	print $OUT_FILE	"$file\t$prob\t$chisum\n";
	#print $OUT_FILE	"@observed\n@predicted\n";
}
```

```
closedir (DIR);
close ($OUT_FILE) or die "Can't close the input file!!!";

################################################################################
# Subroutines
################################################################################
#

# matching_percentage
#
# Subroutine to calculate the percentage of identical bases in two
# equal length DNA sequences
################################################################################

sub matching_percentage {

	my($string1, $string2) = @_;

	# we assume that the strings have the same length
	my($length) = length($string1);
	my($position);
	my($count) = 0;
	my($lengthcount) = 0;

	for ($position=0; $position < $length ; ++$position) {
		if ( (substr($string1,$position,1) =~ /[atcgATCG]{1}/) && (substr($string2,$position,1)
=~ /[atcgATCG]{1}/) ){#ignore gaps and missing data
			++$lengthcount;
			if(uc substr($string1,$position,1) eq uc substr($string2,$position,1)) {
				++$count;
			}
		}
	}


	if ($lengthcount <= 30) {#check if the overlap is longer than 30 bp
		#print "$lengthcount\n";
		return 0;
	}
	else {
		return $count / $lengthcount;
	}

}
```

```
################################################################################
#
# chi_squared_test
#
# Subroutine to calculate the chi-square sum and p values
################################################################################

sub chi_squared_test {
   my ($observed, $expected) = @_;

   my $chi_squared = sum map {
      ($observed->[$_] - $expected->[$_])**2 / $expected->[$_];
   } 0 .. $#$observed;
   my $degrees_of_freedom = @$observed - 1;
   my $probability = chisqrprob($degrees_of_freedom, $chi_squared);
   return ($probability, $chi_squared);
}
```

```
#!/usr/bin/perl -w

##################################################################################
#
# samplegene.pl
#
# select gene file from a folder of fasta files, and concatenate them together
#
# Input: the name of the folder, number of genes want to select, number of repeats want to do.
#
# Output: a directory storing the new fasta files
#
# Written by
#                       Chenhong Li
#                       Shanghai Ocean University, China.
#                       Created on Oct 2015
#                       Last modified at
#
##################################################################################

use warnings;
use strict;
use List::Util qw/shuffle/;

use Getopt::Long;     # include the module for input

my $dir = "aligned"; #variable for the directory storing fasta files
my $outdir = $dir . "selected"; #variable for the output directory
my $numofgenes = 3; #number of genes user want to select
my $numofrep = 1; #number of repeating the gene selection process

my $opt = GetOptions( 'dir:s', \$dir,
                      'outdir:s', \$outdir,
                      'numofgene:s', \$numofgenes,
                      'numofrep:s', \$numofrep,); #set command line options

`mkdir $outdir`; #make output folder


#read all file names from the input foleer
opendir (DIR, $dir) or die $!; #open folder containing all fasta data file
my @filenames;

while (my $file = readdir(DIR)) { #read all fasta files under the folder
```

```
	next if ($file =~ /^\./); #skip files beginning with .
	push (@filenames, "$file");
}
@filenames = sort(@filenames);

#random pick $numofgenes and concatenate them

my $outpath = $outdir . "/$numofgenes" . "gene";
`mkdir $outpath`;
foreach (my $i = 1; $i <= $numofrep; $i++) {#repeat picking genes

my $logfile = "./$outpath/numofgenescaptured.txt";
my $LOGFILE;
open ($LOGFILE, ">$logfile") or die "Can't open the    file!!!";

	my $outfile = $outpath . "/r" . "$i.fas";
	my @subfiles = (shuffle @filenames)[0 .. ($numofgenes - 1) ];
	my $numofgene= cata (\@subfiles, $dir, $outfile);

map {print $LOGFILE "$_\t$numofgene->{$_}\n"} (sort keys %$numofgene);
}

##########################################################################################
# Subroutines
##########################################################################################
# cata
# trim the tree and remove a list of taxa
##########################################################################################

sub cata {
	my $genes = shift @_;
	my @genes = sort(@$genes); #make the array for the list of taxa want to be kept
	my $dir = shift @_;
	my $fasout =    shift @_;

	open OUTFILE, ">$fasout";
	my (%seqlen, %seq, %numofgene);
	foreach my $file (@genes) {
		my $infilename = $dir . "/" . $file;
		open (my $IN_FILE, "<$infilename") or die "Can't open the    file!!!";

		my $seqlength;
		my $lastspecies = ">";
```

```
        while (my $line = readline ($IN_FILE)) {
            chomp $line;
            if (my ($speciesname) = $line =~ /^>(\S+)/) { # if we find >
            $numofgene{$speciesname}++;
                my $nextline = readline ($IN_FILE); #read the sequence in the next line
                chop $nextline;
                if ($speciesname ne $lastspecies) {#only keep the first sequence for each
                    species
                    $seq{$speciesname}->{$file} = $nextline;
                }
                if ($lastspecies eq ">"){
                $seqlength = length($nextline);
                $seqlen{$file} = $seqlength;
                }
                $lastspecies = $speciesname;
            }
        }

        close ($IN_FILE) or die "Can't close the input file!!!";

    }

    foreach my $sample (sort keys %seq) {
    print OUTFILE ">$sample\n";
                    foreach my $gene (sort keys %seqlen){
                        if (!($seq{$sample}->{$gene})){
            my $gaps = '-' x $seqlen{$gene};
                        print OUTFILE $gaps;
                        } else {
                        print OUTFILE $seq{$sample}->{$gene};
                        }
                    }

    print OUTFILE "\n";
    }

close OUTFILE;
return (\%numofgene);
}
```

```
#!/usr/bin/perl -w
####################################################################################
#
# gapdis.pl
#
# calculate the p-distance of individuals
#
# Input: the name of the folder
#
# Output: a folder storing file of each gene's p-distance
#
# Written by
#                           Junning Liu
#                           Shanghai Ocean University, China.
#                           Created on Sep 2016
####################################################################################

use warnings;
use Getopt::Long;      # include the module for input


my $dir = "3gene"; #variable for store the directory
my $outdir = $dir."pdistance"; #variable for store the directory

my $opt = GetOptions( 'dir:s', \$dir,
                                 'outdir:s', \$outdir); #set command line options
  `mkdir $outdir`; #make output folder
opendir (DIR, $outdir) or die $!;
opendir (DIR, $dir) or die $!; #open folder containing all fasta data file
my ($IN_FILE1, $infilename);
      while (my $file = readdir(DIR)) { #read all fasta files under the folder
      next if ($file =~ /^\./); #skip files beginning with .
      my $infilename = "$dir/$file";
      open (my $IN_FILE1, "<$infilename") or die "Can't open the file!!!";
      open ($MY_OUTPUT, ">$infilename.txt");
            while (my $line = readline($IN_FILE1)) {
            chop $line;
            if (($sequencename) = $line =~ /^>(\S+)/) {
                  $seq = readline($IN_FILE1);
                                    chop $seq;
                  }
               $sequence{$sequencename} = $seq;
            }
   my @sequencename = keys(%sequence);
```

```
    #print "@sequencename";
    my @sortedname = sort (@sequencename);
    #print "@sortedname";
    my $sequencenumber = scalar(@sequencename);
    for ($i = 0; $i < $sequencenumber; $i++){
      for ($m = $i+1; $m < $sequencenumber; $m++){
      my $count = 0;
      my $mcount = 0;
      my $flength;
      my $no = "NO";
      my $seq1 = $sequence{$sortedname[$i]};
      my $seq2 = $sequence{$sortedname[$m]};
      my @seq1 = split(//, $seq1);
      my @seq2 = split(//, $seq2);
      my $length = length($seq1);
        for ($n = 0; $n <= $length; $n++){
                                  if ($seq1[$n] eq "-" || $seq2[$n] eq "-"){
                                    $mcount +=1;
                                   } elsif ($seq1[$n] ne $seq2[$n]) {
                                   $count +=1;
                                   }
                                  }

                                $flength = ($length-$mcount);
                                if ($flength == "0") {
       print $MY_OUTPUT "$sortedname[$i]\t$sortedname[$m]\t$no\n" ;
       }
       else {
                                $rate = $count/$flength;
       print $MY_OUTPUT "$sortedname[$i]\t$sortedname[$m]\t$rate\n" ;
                                print "$count, $flength, $length \n";
       }
       }
     }
    close ($MY_OUTPUT);
    close ($IN_FILE1) or die "Can't close the file!!!";
     `mv ./$infilename.txt ./$outdir`;
}
```

```
#!/usr/bin/perl -w
##################################################################################
#
# ID_correct_rate.pl
#
# statistic the right rate of the min p-distance value (if the min p-distance value is intra specific )
#
# Input: the name of the folder,
#
# Output: a dfile storing the new txt file.
#
# Written by
#                    Junning Liu
#                    Shanghai Ocean University, China.
#                    Created on Sep 2016
##################################################################################

use warnings;
use Getopt::Long;    # include the module for input


my $dir = "genepdistance"; #variable for store the directory
my $outfile = "ID_correct_rate.txt"; #variable for store the directory
my $unknown = "CL839_1";
my $unknownsp = "CL839_1 CL839_3 CL839_4 CL839_5 CL839_6";
my $opt = GetOptions( 'dir:s', \$dir,
                         'unknown:s', \$unknown);

opendir (DIR, $dir) or die $!; #open folder containing all fasta data file

my $infilename;
my %hash;
my $count = 0;
my $countfile = 0;
while (my $file = readdir(DIR)) { #read all fasta files under the folder
    next if ($file =~ /^\./); #skip files beginning with .
    $countfile ++;
    my $infilename = "$dir/$file";
    open (my $IN_FILE, "<$infilename") or die "Can't open the file!!!";
                    while (my $line = readline($IN_FILE)) {
                        chomp $line;
                        if ($line =~ s/$unknown\t//g) {
                            my @newline = split /\t/, $line;
                            $hash{$newline[0]} = $newline[1];
```

```
            }
        }

        my @sort_key = sort { $hash{$a} <=> $hash{$b} } keys %hash;
        my @min = grep {$hash{$_} == $hash{$sort_key[0]}} @sort_key;
        $count++ if (map {$unknownsp =~ /$_/} @min);

}

$correct_rate = $count/$countfile;
open (my $OUTFILE, ">$outfile") or die "Can't open the file!!!";
print $OUTFILE "$dir\t$correct_rate\n";
closedir (DIR);
```

```
#!/usr/bin/perl -w

#####################################################################################
#
# pdistance.pl
#
# read the fasta sequences from each file and calculate the p-dist
#
# Input: the directory storing the fasta files .
#
# Output: fasta files by species name
#
# Written by
#                       Chenhong Li
#                       Univeristy of Nebraska - Lincoln, USA.
#                       Created on Nov 2010
#                       Last modified at
#
#####################################################################################

use warnings;


use Getopt::Long;     # include the module for input


my ($dir, $outfile); #variable for store the directory

my $opt = GetOptions( 'dir:s', \$dir,
                        'outfile:s', \$outfile); #set command line options

my ($OUT_FILE); #define output file
open ($OUT_FILE, ">$outfile") or die "Can't open the    file!!!";
print $OUT_FILE "gene\tpdistance\n";


opendir (DIR, $dir) or die $!; #open folder containing all fasta data file
my ($IN_FILE, $infilename);

while (my $file = readdir(DIR)) { #read all fasta files under the folder
    next if ($file =~ /^\./); #skip files beginning with .
    $infilename = "$dir/$file"; #get the name of fasta file
    open ($IN_FILE, "<$infilename") or die "Can't open the    file!!!";
```

```
my @sequence = ();
while (my $line = readline ($IN_FILE)) {
	chop $line; #use chop bc there are maybe hidden characters in dos format files
	if (my ($speciesname) = $line =~ /^>(\S+)/) { # if we find >
		my $nextline = readline ($IN_FILE); #read the sequence in the next line
		chop $nextline;
		push (@sequence, $nextline); #push the squence into the array
	}
}


# Declare and initialize the variables
my $percent;
my @percentages;
my $result;

my $len = length($sequence[0]);

# Iterate through all pairs of sequences
for (my $k = 0 ; $k < scalar @sequence - 1 ; ++$k) {
	for (my $i = ($k + 1) ; $i < scalar @sequence ; ++$i) {

		# Calculate and save the matching percentage
		$percent = matching_percentage($sequence[$k], $sequence[$i]);
		push(@percentages, $percent) if ($percent != 0);
	}
}

# Finally, the average result:
$result = 0;

foreach $percent (@percentages) {
   $result += $percent;
}

$result = $result / scalar(@percentages);
#Turn result into a true percentage
#$result = int ($result * 100);

$result = 1 - $result;

print $OUT_FILE "$file\t$result\n";

close ($IN_FILE) or die "Can't close the input file!!!";
```

```
}

closedir (DIR);
close ($OUT_FILE) or die "Can't close the input file!!!";


################################################################################
# Subroutines
################################################################################
# matching_percentage
#
# Subroutine to calculate the percentage of identical bases in two
# equal length DNA sequences

sub matching_percentage {

	my($string1, $string2) = @_;
	# we assume that the strings have the same length
	my($length) = length($string1);
	my($position);
	my($count) = 0;
	my($lengthcount) = 0;

	for ($position=0; $position < $length ; ++$position) {
		if ( (substr($string1,$position,1) =~ /[atcgATCG]{1}/) && (substr($string2,$position,1)
				=~ /[atcgATCG]{1}/) ){#ignore gaps and missing data
			++$lengthcount;
			if(uc substr($string1,$position,1) eq uc substr($string2,$position,1)) {
				++$count;
			}
		}
	}


	if ($lengthcount <= 30) {#check if the overlap is longer than 30 bp
		print "$lengthcount\n";
		return 0;
	}
	else {
		return $count / $lengthcount;
	}

}
```

```
#!/usr/bin/perl -w

##################################################################################
# concatnexus.pl
#
# read the fasta sequences from each file and concatenate them into a nexus file
#
# Input: the list of species name and the directory storing the fasta files .
#
# Output: a nexus and fasta file containing all data, a txt file containing the number of genes
                captured for each species
#
# Written by
#               Chenhong Li
#               Univeristy of Nebraska - Lincoln, USA.
#               Created on Nov 2010
#               Last modified at
#
##################################################################################

use warnings;


use Getopt::Long;     # include the module for input


my $dir; #variable for store the directory
my $outfile; #variable for store the output file
my $specieslist = "queryOreochromis_niloticus CL964_1 CL964_7 CL964_8 CL964_9
                CL964_10 CL965_2 CL965_3 CL965_4 CL965_5 CL965_7";
my $minnumseq = 1; #the minimum num of seq for a gene being selected

my $opt = GetOptions( 'dir:s', \$dir,
                 'outfile:s', \$outfile,
                 'min:f', \$minnumseq,
                 'specieslist:s', \$specieslist); #set command line options

my %fasta; #hash for storing fasta file

my @list = split/\s/, $specieslist;


opendir (DIR, $dir) or die $!; #open folder containing all fasta data file
my ($IN_FILE, $OUT_FILE, $infilename);
```

```
my $start = 1;
my $end = 1;

$outfile = $outfile . ".nex";
open ($OUT_FILE, ">$outfile") or die "Can't open the output file!!!";

my $numselect = 0; #num of gene selected according to num of seq

my $logfile = $dir . ".numofgenescaptured.txt";
my $LOGFILE;
open ($LOGFILE, ">$logfile") or die "Can't open the    file!!!";

my %genenum;
foreach my $splist (@list) { #open files for output
	$genenum{$splist} = 0;
}

while (my $file = readdir(DIR)) { #read all fasta files under the folder
	next if ($file =~ /^\./); #skip files beginning with .
	$infilename = "$dir/$file"; #get the name of fasta file
	open ($IN_FILE, "<$infilename") or die "Can't open the    file!!!";

	my %seq;
	my $seqlength;
	my $lastspecies = ">";
	while (my $line = readline ($IN_FILE)) {
		chomp $line;
		if (my ($speciesname) = $line =~ /^>(\S+)/) { # if we find >
			$genenum{$speciesname} = $genenum{$speciesname} + 1;
			#if ((my $cleanname) = $speciesname =~ /^([\w\_\d]+[a-zA-Z]{2})[\_\d]+$/) {#if
					species name ended with number
			#	$speciesname = $cleanname;
			#}
			my $nextline = readline ($IN_FILE); #read the sequence in the next line
			if (!$nextline) {#skip first empty line
				#$nextline = readline ($IN_FILE) ;
			}
			chop $nextline;
			if ($speciesname ne $lastspecies) {#only keep the first sequence for each species
				$seq{$speciesname}=$nextline;
			}
			$seqlength = length($nextline) if ($lastspecies eq ">");
			$lastspecies = $speciesname;
		}
```

```
}

close ($IN_FILE) or die "Can't close the input file!!!";


my $numofhash = keys (%seq);
if ($numofhash > $minnumseq){#if the minimum number of sequence
$numselect ++;

	my $gaps = "-";
	for (my $i=1; $i<$seqlength; $i++){
		$gaps .= "-";
	}

	my ($seqid) =    $file =~ /^(\S+)\.aln\.fas/; #get the sequence name
	$end = $start + $seqlength - 1;
	print $OUT_FILE "[$seqid $seqlength bp $start-$end bp]\n";
	$start = $end + 1;

	foreach my $list (@list) { #open files for output
		print $OUT_FILE "$list\t";
		if ($seq{$list}){
			print $OUT_FILE $seq{$list};
			print $OUT_FILE "\n";
			if ($fasta{$list}){
				$fasta{$list} = $fasta{$list} . $seq{$list};
			}
			else{
				$fasta{$list} = $seq{$list};
			}

		}
		else{
			print $OUT_FILE "$gaps\n";
			if ($fasta{$list}){
				$fasta{$list} = $fasta{$list} . $gaps;
			}
			else{
				$fasta{$list} = $gaps;
			}
		}
	}

}
```

```
	print $OUT_FILE "\n";

}

closedir (DIR);

close ($OUT_FILE) or die "Can't close the outfile!!!";

foreach my $splist (@list) { #open files for output
	print $LOGFILE "$splist\t$genenum{$splist}\n";
}


my ($FASTA_OUTFILE);
my $fasout = $outfile . ".fas";

open ($FASTA_OUTFILE, ">$fasout") or die "Can't open the output file!!!";

foreach my $list (@list) { #open files for output
	print $FASTA_OUTFILE ">$list\n$fasta{$list}\n";
}


close ($FASTA_OUTFILE) or die "Can't close the outfile!!!";

print "\nThere are $numselect genes included in the output files!\n\n";
```
